# Differentiation and validation of mild and severe strains of citrus tristeza virus through codon usage bias, host adaptation, and biochemical profiling

**DOI:** 10.3389/fmicb.2025.1665893

**Published:** 2025-10-01

**Authors:** Halima Khatoon, Dharmappa D. Chavan, Vijay Kamal Meena, Abhijeet Shankar Kashyap, Kumar Sathiyaseelan, Marimuthu Elangovan, Lalit Patil, Bharat Raj Meena, Aarti Gauns, Utpal Kumar Bhattacharyya, Kajal Kumar Biswas

**Affiliations:** ^1^Advanced Centre for Plant Virology, Division of Plant Pathology, ICAR-Indian Agricultural Research Institute, New Delhi, India; ^2^Agriculture university Jodhpur (Agricultural Research Sub-Station, Sumerpur), Jodhpur, India; ^3^ICAR- National Bureau of Agriculturally Important Microorganism, Mau, Uttar Pradesh, India; ^4^Goa College of Agriculture, Ella Farm Old Goa, India; ^5^ICAR-Regional Centre for North Eastern Hill Region, Kanubai, India

**Keywords:** coat protein, codon usage bias, codon adaptation index (CAI), principal component analysis (PCA), biological indexing

## Abstract

Citrus tristeza virus (CTV) is one of the most economically significant citrus pathogens, causing epidemics worldwide. It comprises strains ranging from asymptomatic mild variants to highly virulent severe forms. However, the molecular basis distinguishing mild from severe strains remains poorly characterized. To understand these mechanisms, a total of fifty eight citrus samples were screened using RT-PCR targeting the coat protein gene, with forty seven testing positive for CTV. Analysis of Codon Usage Bias (CUB) revealed that both mutation pressure and natural selection influence codon preferences, with natural selection playing the dominant role. Hierarchical clustering dendrogram based on Effective Number of Codons (ENc) values reveals twenty mild-like isolates and twenty seven severe strains. Among the mild-like group, nine mild strains exhibited higher Codon Adaptation Index with uracil at the third codon position and Relative codon usage patterns closely aligned with the Host, suggesting greater translational efficiency and reduced virulence. Quantitative RT-PCR confirmed lower viral accumulation in plants infected with mild strains, with a ninefold increase observed in those infected by severe strains. Validation through biological indexing confirmed the mild nature of these isolates and their ability to confer cross-protection upon challenge with severe strains. Phylogenetic relationships and sequence identity metrics indicated a close genetic association of mild strains with the VT strain, highlighting their genetic relatedness. Additionally, biochemical profiling revealed distinct patterns in sugar, phenol, antioxidant, and chlorophyll levels across mild, severe, and healthy plants. These findings highlight the promise of mild, well-adapted CTV strains as effective agents for cross-protection in citrus orchards.

## 1 Introduction

Citrus is one of the most economically significant fruit crops globally, cultivated across diverse agroecological zones for its rich nutritional value and recognized health benefits (Liu et al., 2012). With an annual production of approximately 161.8 million tons grown on 10.2 million hectares worldwide, it ranks as the second most widely cultivated fruit crop (FAO, 2023). However, citrus cultivation is increasingly threatened by a range of biotic and abiotic stresses, among which viral diseases, especially those caused by citrus tristeza virus (CTV), pose a major challenge. CTV is a phloem-limited pathogen responsible for widespread tree decline, stem pitting, and substantial reductions in fruit yield and quality, resulting in significant economic losses in key citrus-producing regions such as Brazil, Argentina, California, and Venezuela (Rocha-Peña et al., 1995; Ahlawat and Pant, 2003).

CTV belongs to the genus *Closterovirus* within the family *Closteroviridae*. It possesses long, flexuous virions (10–12 nm × 2000 nm) and a monopartite, positive-sense single-stranded RNA genome of ~19.3 kb that encodes 12 open reading frames (ORFs) (Bar-Joseph et al., 2002). The 5′ proximal ORF1a encodes a large polyprotein (~349 kDa) with methyltransferase and helicase-like domains, while downstream ORFs are expressed through subgenomic RNAs (Karasev et al., 1995; Hilf et al., 1995). These encode proteins involved in virion assembly (CP, CPm, p. 65, p. 61), movement (p. 6), and suppression of host RNA silencing (CP, p. 20, p. 23). Notably, the CP (ORF7) encodes the major coat protein (~25 kDa) that encapsidates ~95% of the virion, while the minor coat protein CPm (ORF6) covers the remaining 5% (Febres et al., 1996).

CTV exhibits substantial genetic variability across and within regions. Eight major phylogroups, T30, T36, T3, T68, VT, RB, HA16-5, and S1, have been described based on whole-genome analyses (Roy and Brlansky, 2010; Melzer et al., 2010; Harper, 2013; Yokomi et al., 2018). Mild or asymptomatic strains such as T30, S1, and RB are often contrasted with severe variants like VT, T68, and T3, which cause significant pathogenic symptoms (Albiach-Marti, 2013; EPPO, 2023). In India, strains such as Kpg3 have been identified as decline-inducing and form the same clade with VT (Biswas et al., 2012).

Effective management of CTV has involved various strategies, including quarantine enforcement, certified virus-free budwood programs (Kyriakou et al., 1996; Navarro et al., 2002), eradication of infected trees (Bar-Joseph et al., 1989; Gottwald et al., 2002), deployment of tolerant rootstocks (Forner-Giner et al., 2009; Guarino et al., 2021), and transgenic approaches (Cheng et al., 2015). However, these methods are often labor-intensive, time-consuming, and limited in scalability. Moreover, chemical control of aphid vectors poses ecological and health risks due to pesticide resistance and non-target effects.

In this context, integrated and sustainable approaches such as Mild Strain Crop Protection (MSCP) are gaining prominence. MSCP merges ecological principles with precision agriculture to improve crop resilience while reducing dependence on water, fertilizers, and synthetic chemicals (Foley et al., 2011; Van Bueren et al., 2018). Among such sustainable solutions, MSCP has emerged as a promising method for managing viral diseases. This strategy involves pre-inoculating plants with a mild strain of the virus, which can effectively prevent or suppress subsequent infections by severe strains of the same virus (Lee and Keremane, 2013). Folimonova et al. (2010) demonstrated that infection with a CTV strain can exclude subsequent infection by another isolate of the same strain. MSCP is a phenomenon that has enabled continued citrus cultivation in regions affected by severe CTV isolates (Fraser et al., 1968; Costa and Müller, 1980; Vance and Vaucheret, 2001; Müller and Rezende, 2004).

Understanding the molecular and evolutionary mechanisms that differentiate mild from severe CTV strains is critical for optimizing MSCP. One such mechanism is Codon Usage Bias (CUB) the non-random usage of synonymous codons in coding sequences. Since viruses rely on host translational machinery, their codon usage often evolves in tandem with that of their host to enhance translation efficiency and viral fitness (Burns et al., 2006; Chaney and Clark, 2015; Kumar et al., 2016). CUB has been linked to replication efficiency, host adaptation, and immune evasion (Plotkin and Kudla, 2011; Stoletzki and Eyre-Walker, 2007; Costafreda et al., 2014), and co-evolutionary trends in CUB have been reported in various plant RNA viruses (Franzo et al., 2017; Rahman et al., 2017; Simón et al., 2017; Adams and Antoniw, 2003; Xu et al., 2008; Cheng et al., 2012; Chakraborty et al., 2015). Although previous research has addressed aspects of host-CTV interaction and viral adaptation (Cheng et al., 2012; Biswas et al., 2019), it has not attempted to identify specific mild strains or conduct detailed comparisons between mild and severe CTV strains in terms of codon usage and host adaptation mechanisms leaving a critical gap in our understanding of the molecular basis underlying the asymptomatic nature of mild strains in contrast to the pathogenicity exhibited by severe strains.

In addition to genetic factors, viral infections alter host physiology and biochemical responses. Plants, including citrus, employ a wide array of antioxidant mechanisms-both enzymatic and non-enzymatic-to mitigate oxidative stress during pathogen attack (Lattanzio et al., 2006; Gorinstein et al., 2004). These include phenolic compounds, ascorbate, and antioxidant enzymes such as peroxidases and superoxide dismutase (Riedle-Bauer, 2000; Hernández et al., 2004; Ashfaq et al., 2014; Wang et al., 2021; Jyoti et al., 2023). However, the biochemical responses of citrus plants to mild versus severe CTV strains remain poorly characterized, limiting our ability to identify biochemical markers for strain differentiation or resistance.

Therefore, the present study aims to systematically investigate the molecular, evolutionary, and biochemical distinctions between mild and severe strains of CTV in *Citrus reticulata*. Using 47 field-collected isolates maintained under controlled conditions at ICAR-IARI, New Delhi, this work applies codon usage analysis (CAI, RCUB, PCA), viral titer quantification via qRT-PCR, biological indexing, and detailed biochemical profiling to elucidate host-virus interactions. The insights derived will inform the strategic identification of candidate mild strains for future cross-protection programs and contribute to sustainable CTV management strategies.

## 2 Materials and methods

### 2.1. Dataset

A total of fifty eight plant RNA samples were isolated from the bark tissues and petioles of infected and healthy citrus plants using the RNeasy Plant Mini kit (Qiagen) following the manufacturer's instructions. Finally, the isolated RNA was eluted in 20 μl of water. First-strand cDNA was generated according to the methodology employed by Biswas (2008). Two primer sets, CP11F and CP11R, were utilized to identify the CTV coat protein gene via RT-PCR. PCR was conducted in a Thermal cycler (Bio-Rad) under the following temperature parameters: a 5-min hot start at 94 °C, followed by 35 cycles including denaturation at 94 °C for 30 sec, annealing at 59 °C for 35 sec, and extension at 72 °C for 45 sec. The final extension occurred at 72 °C for 10 min (Supplementary Table 1). The PCR results were analyzed using a 1% agarose gel produced in 1X TAE buffer with 200 ng of Ethidium bromide/ml.

CP gene of isolates from the present study, along with 60 Indian isolates and 6 globally recognized CTV genotypes VT, T36, B165, T3, NZRB-G90, and T30, were taken for the study of CUB in the present study (Supplementary Table 2). The CP genes were taken for CUB analysis, as the highest degree of codon adaptation was observed for the CP genes because these genes are expressed at high levels in the plant cells. The origin of 58 present CTV isolates assayed and their background were given in (Supplementary Table 3).

### 2.2. Sequence analysis

The corresponding sequence of international VT, T36, T30, B165, NZ-RB, and HA16-5 recognized internationally (Melzer et al., 2010; Roy and Brlansky, 2010; Biswas et al., 2012) and previously reported Indian isolates (Biswas, 2010; Tarafdar et al., 2013) were used for sequence comparison of present isolates. The multiple sequence alignments were carried out using Clustal X version 1.81 (Thompson et al., 1997). Maximum likelihood phylogenetic trees were constructed using software MEGA11 (Tamura et al., 2021). A sequence identity matrix was generated using the sequence demarcation tool (SDT) version 1.2 (Muhire et al., 2014).

### 2.3 Nucleotide composition analysis and effective number of codons (ENc)

The nucleotide composition parameters, including the overall frequencies of A%, U%, C%, and G%, as well as the nucleotide frequencies at the third (wobble) position of synonymous codons (A3%, U3%, C3%, and G3%), were calculated for the CP gene sequences of each CTV isolate. Additionally, the G+C content at the first (GC1), second (GC2), and third (GC3) codon positions, along with the combined G+C content at the first and second positions (GC1, 2), were determined using CodonW version 1.4.2 and the web server http://genomes.urv.es. The ENc values were employed to assess the extent of codon usage bias (CUB) in the CP gene. ENc values typically range from 20 to 61, where 20 indicates extremely strong codon bias, while 61 signifies equal usage of all synonymous codons. An ENc value of 35 or lower indicates strong codon usage bias.

### 2.4 ENc-gc3 plot and neutrality plot

An ENc-GC3 plot was employed to analyze the impact of mutation and natural selection on CUB. If mutation predominantly influences CUB, the ENc values are expected to align closely with the standard curve. Conversely, if natural selection plays a significant role, the ENc values will deviate substantially below the standard curve. This method effectively distinguishes between mutational pressure and selective constraints in shaping codon usage patterns.

A neutrality plot is a valuable tool for assessing the influence of mutational constraints and natural selection on viral genes. The slope of the regression line plays a crucial role in interpreting these effects. In neutrality plots, a statistically significant correlation between GC12 and GC3, along with a regression line slope close to 1 (indicating points aligned along the diagonal), suggests that mutation pressure predominantly influences CUB. Conversely, a weak or absent correlation between GC12 and GC3 indicates that selection plays a greater role in counteracting mutation bias. This approach provides important insights into the evolutionary dynamics of viral genomes.

### 2.5 Identification of putative mild CTV isolates through CUB analysis

The resulting ENc values were subjected to hierarchical clustering to group isolates based on similarities in their codon usage patterns. Clustering was performed in R software (version 4.2.2) using the hclust function from the *stats* package. The Euclidean distance matrix was calculated, and the complete linkage method was applied for tree construction. The dendrogram was visualized using the tidy verse for data wrangling and Factoextra for cluster visualization. Clusters were annotated based on the known biological classification of reference strains: isolates clustering with T30-type (a known mild strain) were considered putative mild strains, while those clustering with VT-type (associated with severe symptoms) were considered putative severe strains. This approach allowed preliminary classification of isolates based on CUB. These putative mild strains will be further confirmed through other indices.

#### 2.5.1 Codon Adaptation Index (CAI)

The Codon Adaptation Index (CAI) is a quantitative metric used to predict the degree of adaptation of viruses to their potential host. CAI values are calculated using the web server http://genomes.urv.es/CAIcal/. The index ranges from 0 to 1, with higher CAI values indicating a stronger preference for specific codon usage patterns, suggesting better adaptation compared to sequences with lower CAI values.

#### 2.5.2 Relative synonymous codon usage (RSCU)

The Relative Synonymous Codon Usage (RSCU) value represents the ratio of a codon's observed frequency to its expected frequency, assuming equal usage of all codons for a given amino acid. RSCU values less than 1.0 indicate negative codon usage bias, while values greater than 1.0 reflect positive bias, with RSCU = 1.0 signifying no bias. The RSCU values for viruses and hosts were calculated following a previously described method, as shown in the equation below.


$$\begin{array}{l} RSCU_{ij}=\;\frac{g_{ij}}{\overset{ni}{\underset{j}{\sum }}g_{ij}}\times ni \end{array}$$

#### 2.5.3 Principal component analysis (PCA)

To assess the overall codon usage variation between the host (*Citrus reticulata*) and different strains of CTV, a PCA was conducted. PCA is a statistical technique used to reduce the dimensionality of complex datasets. It helps in identifying patterns such as clusters or gradients within the data. Analyzing the contribution of original numerical variables to the principal components also provides valuable insights. To explore potential associations between the principal axes and compositional features, Pearson correlation tests were conducted. PCA was carried out in R using the pr.comp function. Visualizations were generated using the scatter 3D plot package (Ligges, 2003).

#### 2.5.4 Quantitative RT-PCR (qpcr) assay for the viral load in leaf tissues CTV

Quantitative real-time PCR (qRT-PCR) was conducted to determine the viral load in systemic leaf tissues of plants infected with mild and severe strains. Total RNA was isolated from 100 mg of plant tissue using the SV Total RNA Isolation System (Promega, USA) and eluted in 20 μL of elution buffer. RNA quantity and quality were evaluated using a Nabi–Microdigital NanoDrop spectrophotometer (Genetix Biotech Asia Pvt. Ltd.). For cDNA synthesis, 500 ng/μL of total RNA was utilized, which subsequently served as the template for qRT-PCR analysis. Viral load quantification was performed using qRT-PCR with specific primers (118F/118R) targeting the CP gene of CTV, and the citrus actin gene (Supplementary Table 4) was used as an internal control for normalization. DNA concentrations were adjusted to 90 ng/μL to ensure consistent amplification efficiency. The qRT-PCR reactions were set up using SYBR^^®^^ Green PCR Master Mix (Applied Biosystems) and run on a Bio-Rad CFX96 Touch Real-Time PCR Detection System. The thermal cycling protocol included an initial denaturation at 95 °C for 3 min, followed by 35 cycles of denaturation at 95 °C for 30 sec, annealing at 58 °C for 30 sec, and extension at 72 °C for 30 sec. This was followed by five additional cycles of denaturation at 95 °C for 5 sec, annealing at 64 °C for 5 sec, and a final extension at 95 °C for 5 min. Each biological sample was analyzed in triplicate to ensure reproducibility and accuracy. Viral load was calculated using the 2^−^ΔΔCt method (Livak and Schmittgen, 2001), with the mild strain-infected rootstock serving as the reference for relative expression analysis.

### 2.6 Biological indexing of putative mild strain.

Seedlings were raised using healthy seeds collected from the Division of *Fruits* and Horticultural Technology, ICAR-IARI, New Delhi. Fruits were rinsed in 2% sodium hypochlorite before seed extraction to avoid unwanted contamination. Afterwards, disinfect the extracted seeds and thoroughly rinse them with water. The seeds were then rinsed with distilled water and spread evenly on non-stick paper to dry completely, avoiding direct sunlight. Dried seeds are stored in polyethene bags at 4–10 °C. The seeds were sown in earthen pots of 12-inch diameter containing field soil and farm yard manure in a ratio of 1:1. The seedlings were transplanted singly in earthen pots of 8-inch diameter and maintained in the insect-proof chambers with spraying Metasystox 25 EC (0.05%) at 15-days intervals. Biological indexing was performed to assess symptom expression of putative mild CTV strains. Seedlings of Rough lemon (*Citrus jambhiri*) and Kagzi lime (*Citrus aurantifolia*) were graft-inoculated with the following mild strains: DeKM-2, ASKM-1, ASKM-2, DeKM-8, and DeKM-5, with three replicates per treatment. Severe CTV strains (Kpg-3) were included as controls, also with three replicates, under identical conditions. Plants were maintained in a greenhouse, and symptom development was recorded weekly for 6–8 weeks. The mild nature of the selected strains was further confirmed through challenge inoculate with severe strain.

### 2.7 Biochemical analysis between the mild and severe strain

To compare the biochemical responses induced by mild and severe CTV strains, leaf samples were collected from seedlings of Kagzi lime graft-inoculated with the respective strains. The putative mild strains used were DeKM-2, ASKM-1, DeKM-8, and DeKM-5, while the severe strains included Kpg-3, GDKM-1, and BHKM-9. Sampling was performed at 7 weeks post-inoculation, with three biological replicates per treatment. Collected leaf samples were immediately frozen in liquid nitrogen and stored at −80 °C until further biochemical analyses. The analyses included assessment of parameters such as (Total antioxidant, Total phenolic content, Total chlorophyll content, and Total sugar) to evaluate the differential responses induced by mild versus severe CTV strains.

#### 2.7.1 Total sugar

The total soluble sugar content was estimated using the Anthrone method as described by David T. (Plummer 1990). This method relies on the dehydration of carbohydrates by sulfuric acid, forming furfural, which reacts with anthrone to produce a blue-green complex measurable at 630 nm.A 0.1 g sample was homogenized in 10 mL of 80% ethanol, centrifuged at 5000 g for 20 min, and the supernatant was collected. The residue was re-extracted twice with 5 mL of 80% ethanol, and the combined supernatants were adjusted to 25 mL. A 100 μL aliquot was evaporated in a boiling water bath, cooled, and dissolved in 1 mL of water before adding 4 mL of anthrone reagent. The mixture was then heated in a boiling water bath for 8 min, and the absorbance was measured at 630 nm. The carbohydrate content was determined using a standard curve and expressed as a percentage.


$$\begin{array}{l} \text{Amount of carbohydrate present in sample }\left(\%\right)\;=\\ \frac{Sugar\;value\;from\;graph\;\left(mg\right)\times Total\;volume\;of\;extract\left(ml\right)}{Aliquot\;sample\;used\times wt\;of\;sample\left(mg\right)}\\ \times 100 \end{array}$$

#### 2.7.2 Total antioxidants estimation

The antioxidant capacity of the samples was evaluated using the Ferric Reducing Antioxidant Power (FRAP) assay, as described by Benzie and Strain (1999). This method measures the reduction of the Fe^3+^-TPTZ complex to its blue Fe^2+^ form in the presence of antioxidants under acidic conditions.

The FRAP reagent was prepared by combining TPTZ (40 mmol/L in HCl), FeCl3 (20 mmol/L), and acetate buffer (0.3 mol/L, pH 3.6), followed by warming to 37 °C. A 40 μL sample aliquot was mixed with 0.2 mL of distilled water and 1.8 mL of the FRAP reagent, then incubated at 37 °C for 15 min. Absorbance was recorded at 593 nm using a spectrophotometer.A standard curve was prepared using 1 mmol/L FeSO4, and the antioxidant capacity was expressed as FeSO4 equivalents, with appropriate dilutions performed as required.

#### 2.7.3 Total phenol estimation

Total phenolic content was determined using the Folin-Ciocalteu reagent method described by Malik and Singh (1980). This method reduces a phosphotungstate-phosphomolybdate complex by phenolic compounds, producing a blue-colored product. The absorbance of the resulting mixture was measured at 760 nm against a blank, with gallic acid serving as the standard. A standard curve of gallic acid was prepared to quantify the phenolic content in the samples, which was expressed as gallic acid equivalents (mg/mL).


$$\begin{array}{l} \text{Total phenol }\left(\text{mg}/100\text{g GAE}\right)\;=\\ \frac{Total\;volume\;of\;extract\times Conc.\;of\;phenol}{Weight\;of\;sample\times amount\;of\;aliquot\times \;10000} \end{array}$$

#### 2.7.4 Total chlorophyll concentration

The Chlorophyll Concentration Index (CCI) was measured using the MC-100 Chlorophyll Concentration Meter (Apogee Instruments). This non-destructive method estimates chlorophyll content by measuring light transmission through leaf tissue at specific wavelengths. The MC-100 device calculates CCI based on the difference in absorbance between wavelengths strongly absorbed by chlorophyll and those with minimal absorption. This index provides a reliable estimate of chlorophyll concentration, reflecting the plant's physiological status and overall health.

#### 2.7.5 Statistical analysis

A one-way analysis of variance (ANOVA) was carried out using SPSS software to evaluate differences between mild, severe and healthy. To identify significant differences between group means, Tukey's Honestly Significant Difference (HSD) test was applied as a post hoc analysis. Additionally, the standard error of the mean (SE) was calculated in SPSS to measure the variability of the data.

## 3 Result

### 3.1. Dataset

**Figure 1:**
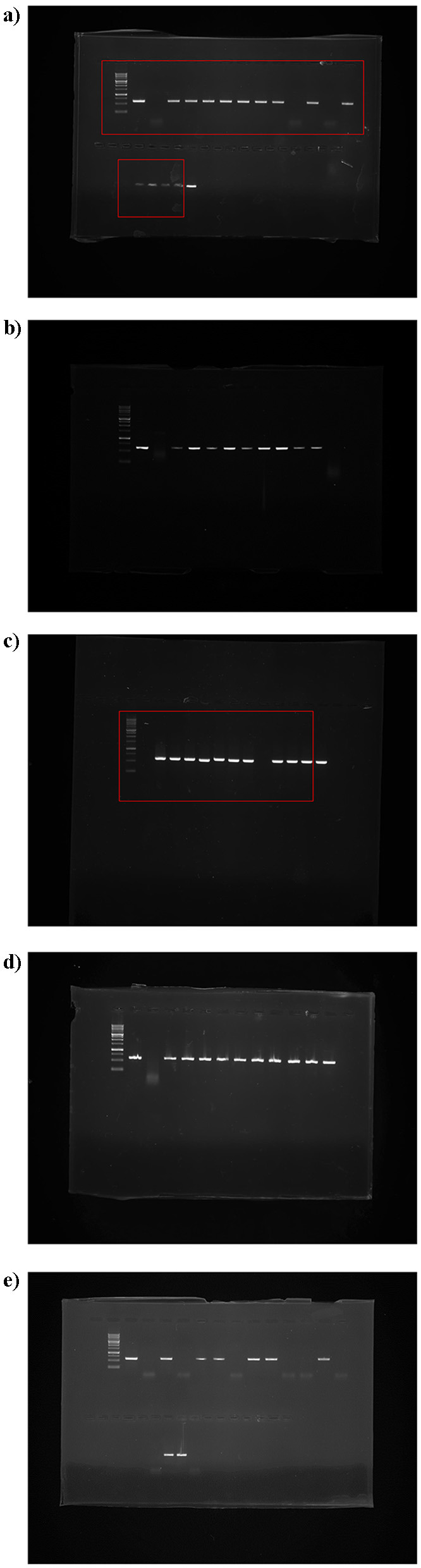
Agarose gel electrophoresis showing amplification of 672 bp genome from 3′ variable CP region of CTV using specific oligonucleotide primers by RT-PCR, with lane M: 1kb ladder, Lane +ve: positive control, Lane –ve: negative control; **(a)** Kamrup Metro samples (lanes 1–15); **(b)** Kamrup Rural samples (lanes 1–8); **(c–e)** Goalpara samples (c: lanes 1–10, d: lanes 1–10, e: lanes 1–15). **(a)**, lanes from the lower section of the original gel were digitally repositioned above for improved comparative alignment. A clear vertical splice mark has been included to indicate this adjustment. All other gel images are presented as in the original, without any modifications).

Out of the 58 citrus samples tested, 47 were positive for the CTV coat protein CP gene, while 11 samples were CTV free, indicating the absence of the target viral sequence (Figure 1). The RT-PCR amplification using the CP11F and CP11R primer set produced a distinct band of approximately 672 base pairs, corresponding to a partial fragment of the 3′ variable region of the CTV CP gene. (For Figures 1a–e, lanes from the lower section of the original gel were digitally repositioned above for improved comparative alignment. A clear vertical splice mark has been included to indicate this adjustment. All other gel images are presented as in the original, without any modifications).

### 3.2. Natural selection and mutation pressure contribute to the codon usage bias observed in CTV

**Figure 2:**
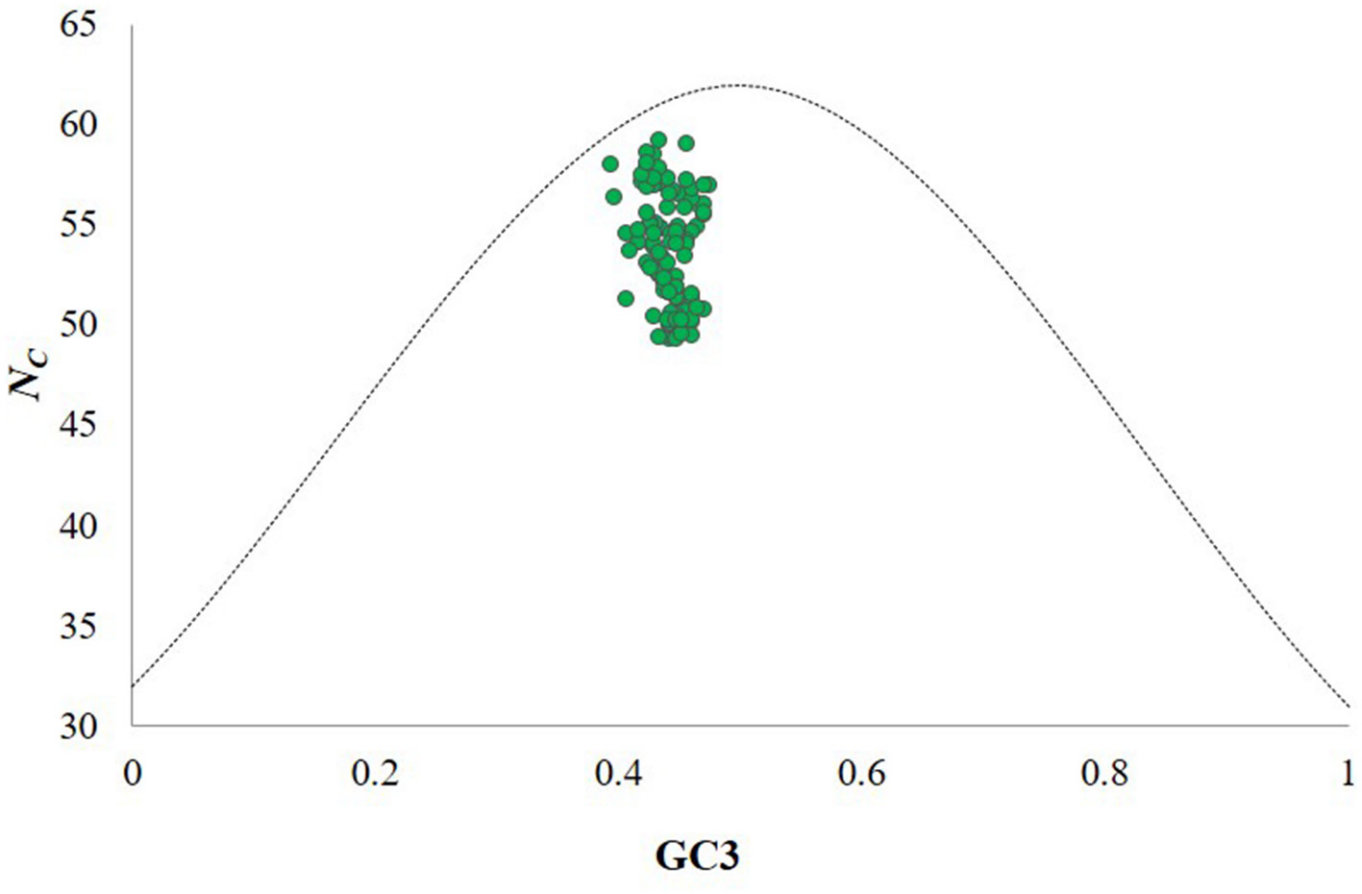
ENc-GC3 plot analysis of the CP genes of CTV isolates. ENc denotes the effective number of codons, and GC3 denotes the GC content at the third synonymous codon position. The green dotted line represents the expected curve derived from the positions of strains when the codon usage is only determined by the GC3 composition. CTV isolates indicate that the codon choice selection of CTV is influenced by translational selection, gene length and mutation bias. The dotted curve indicates the expected curve when all codons are used randomly (no selection).

An ENc-GC3 plot was constructed with ENc values on the y-axis and GC3 values on the x-axis to assess the impact of mutation and natural selection on CUB (Supplementary Table 5). Each point in the plot corresponds to the CP gene of an individual CTV isolate. The dotted curve represents the expected distribution if codons were used randomly. Notably, the GC3 content values of the CP genes for all CTV isolates fell below this dotted curve and the ENc values of CTV isolates ranged from 49.29 to 59.2, with a mean of 53.96 ± 0.22 at GC3 values of 0.31–0.42 (Figure 2) suggesting that codon usage in the CP gene of CTV is influenced by both natural selection and mutational pressure.

### 3.3 Natural selection plays a crucial role in shaping the codon usage bias of CTV

**Figure 3:**
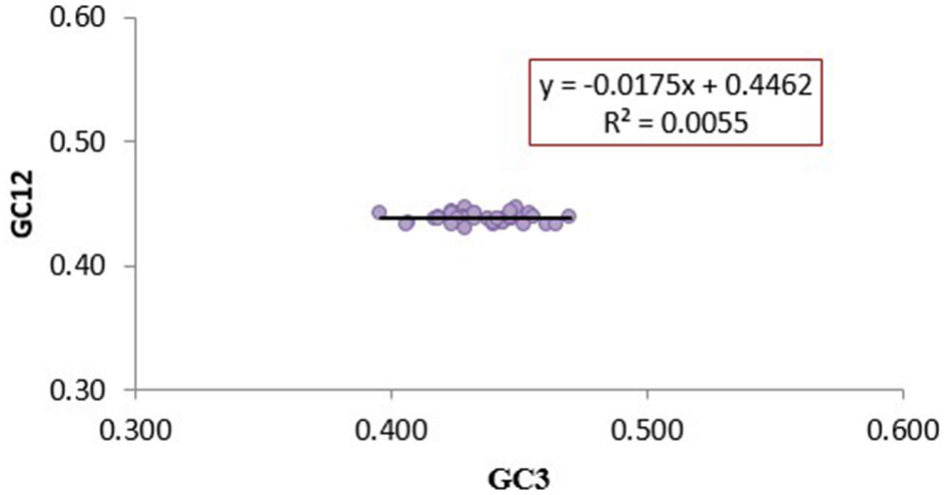
Neutrality plot analysis (GC1 and GC2 vs. GC3) for the coat protein genes of CTV isolates. GC1 and GC2 stand for the average value of GC contents at the first and second positions of the codons, while GC3 refers to the GC contents at the third position of the codons. The Color marker is the linear regression of GC1 and GC2 against GC3; the regression curve can be described as *y* = −0.0175x +0.4462, *R*^2^ = 0.0055.

The magnitude of natural selection and mutation pressure in CUB was examined using a neutrality plot (GC12 vs. GC3). The slope of the regression line (y = −0.0175x +0.4462) was close to zero, with an R^2^ value of 0.0055. The narrow range of GC3 values (0.39–0.47) and the absence of a significant correlation between GC12 and GC3 indicate minimal influence of mutation pressure (Figure 3). This weak correlation suggests that natural selection predominantly shapes the codon usage pattern in CTV's analyzed CP genes.

### 3.4 Nucleotide compositional constraints significantly influence codon usage bias in CTV

The relationship between CUB and nucleotide composition was analyzed through correlation analysis (Table 1). The analysis revealed that the ENc showed a significant positive correlation with A3s and C3s (*r* = 0.605 and *r* = 0.545, respectively, at *p* < 0.01) and a significant negative correlation with U3s (*r* = −0.547 at *p* < 0.01). This suggests that an increase in U at the third nucleotide position is associated with a decrease in ENc, indicating higher codon usage bias.

**Table 1:** Correlation analysis among different nucleotide compositions of CTV.

**Indices**	**U3s**	**C3s**	**A3s**	**G3s**	**CAI**	**CBI**	**Fop**	**Nc**	**GC1s**	**GC2s**	**GC12**	**GC3s**	**GC**	**L_sym**	**L_aa**	**Gravy**	**Aromo**
T3s	1																
C3s	−0.55^**^	1															
A3s	−0.563^**^	0.325^**^	1														
G3s	0.318^**^	−0.367^**^	−0.908^**^	1													
CAI	0.371^**^	0.088	0.133	−0.284^**^	1												
CBI	0.117	0.321^**^	−0.194^*^	0.096	−0.389^**^	1											
Fop	0.334^**^	0.374^**^	−0.214^*^	0.024	0.044	0.896^**^	1										
Nc	−0.547^**^	0.545^**^	0.605^**^	−0.596^**^	0.157	−0.099	−0.08	1									
GC1s	−0.038	0.257^**^	0.208^*^	−0.225^*^	0.255^**^	0.081	0.172	0.328^**^	1								
GC2s	−0.115	−0.026	−0.015	0.022	0.027	−0.239^**^	−0.236^*^	0.151	−0.12	1							
GC12	−0.107	0.199^*^	0.165	−0.175	0.232^*^	−0.087	−0.008	0.374^**^	0.764^**^	0.549^**^	1						
GC3s	−0.125	0.197^*^	−0.715^**^	0.821^**^	−0.355^**^	0.233^*^	0.127	−0.256^**^	−0.12	0.066	−0.058	1					
GC	−0.71^**^	0.027	0.057	0.167	−0.627^**^	−0.256^**^	−0.578^**^	0.14	−0.108	0.162	0.015	0.364^**^	1				
L_sym	0.527^**^	0.19^*^	−0.303^**^	0.174	0.554^**^	0.276^**^	0.587^**^	−0.174	0.215^*^	0.074	0.229^*^	0.135	−0.763^**^	1			
L_aa	0.521^**^	0.193^*^	−0.295^**^	0.171	0.556^**^	0.27^**^	0.582^**^	−0.172	0.217^*^	0.073	0.23^*^	0.133	−0.762^**^	1^**^	1		
Gravy	−0.365^**^	−0.184^*^	0.103	−0.042	−0.631^**^	−0.105	−0.412^**^	0.09	−0.255^**^	0.021	−0.201^*^	−0.028	0.603^**^	−0.753^**^	−0.76^**^	1	
Aromo	−0.584^**^	−0.153	0.367^**^	−0.217^*^	−0.569^**^	−0.294^**^	−0.607^**^	0.208^*^	−0.269^**^	0.053	−0.193^*^	−0.162	0.753^**^	−0.913^**^	−0.913^**^	0.806^**^	1

^**^Correlation is significant at the 0.01level (two-tailed); ^*^correlation is significant at the 0.05 level (two-tailed).

### 3.5 Codon usage bias has significant correlation with the nucleotide compositional constraint in CTV

**Figure 4:**
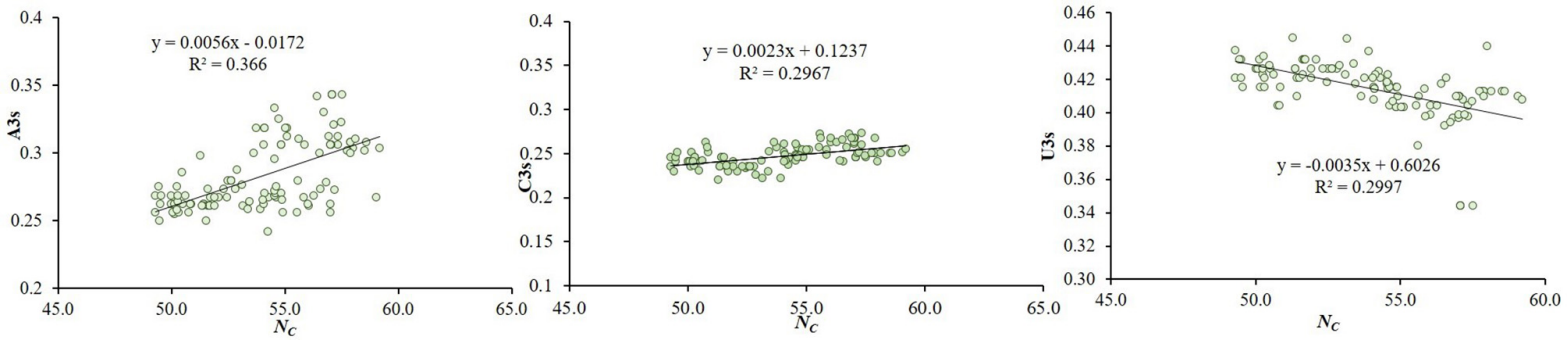
Correlations between ENc and the nucleotide composition of the CTV coat protein gene. The relationship of ENc with correlation with A3s, U3s and C3s (regression curves value of a: A3(*y* = 0.0056x−0.0172 *R*^2^ = 0.36); b: C3(*y* = *y* = 0.0023x + 0.1237, *R*^2^ = 0.2967), c: U3 (*y* = −0.0035x + 0.6026, *R*^2^ = 0.2997) at *p* < 0.01), suggesting that an increasing Uracil composition may enhance the CUB that is influenced by the nucleotide compositional constraint in CTV.

The relationship between CUB and nucleotide composition was investigated through multivariate correlation analysis. In the analysis, ENc showed significant positive correlation with C3(*y* = 0.0023x + 0.1237, *R*^2^ = 0.2967), and: A3(*y* = 0.0056x−0.0172 *R*^2^ = 0.36 *p* < 0.01) and significant negative correlation with U3(*y* = −0.0035x + 0.6026, *R*^2^ = 0.2997 at *p* < 0.01) (Figure 4). It suggests that an increasing U nucleotide composition may enhance the CUB, which is influenced by the nucleotide.

### 3.6 Identification of putative mild CTV isolates through CUB analysis

The magnitude of CUB in the CP gene of 117 CTV isolates was assessed using the ENc. The ENc values among these CTV isolates were relatively high, ranging from 49.29 to 59.2, with a mean of 53.96 ± 0.22 (Supplementary Table 5). These higher ENc values indicate low codon usage bias in the CP genes, suggesting greater genomic stability in CTV.

**Figure 5:**
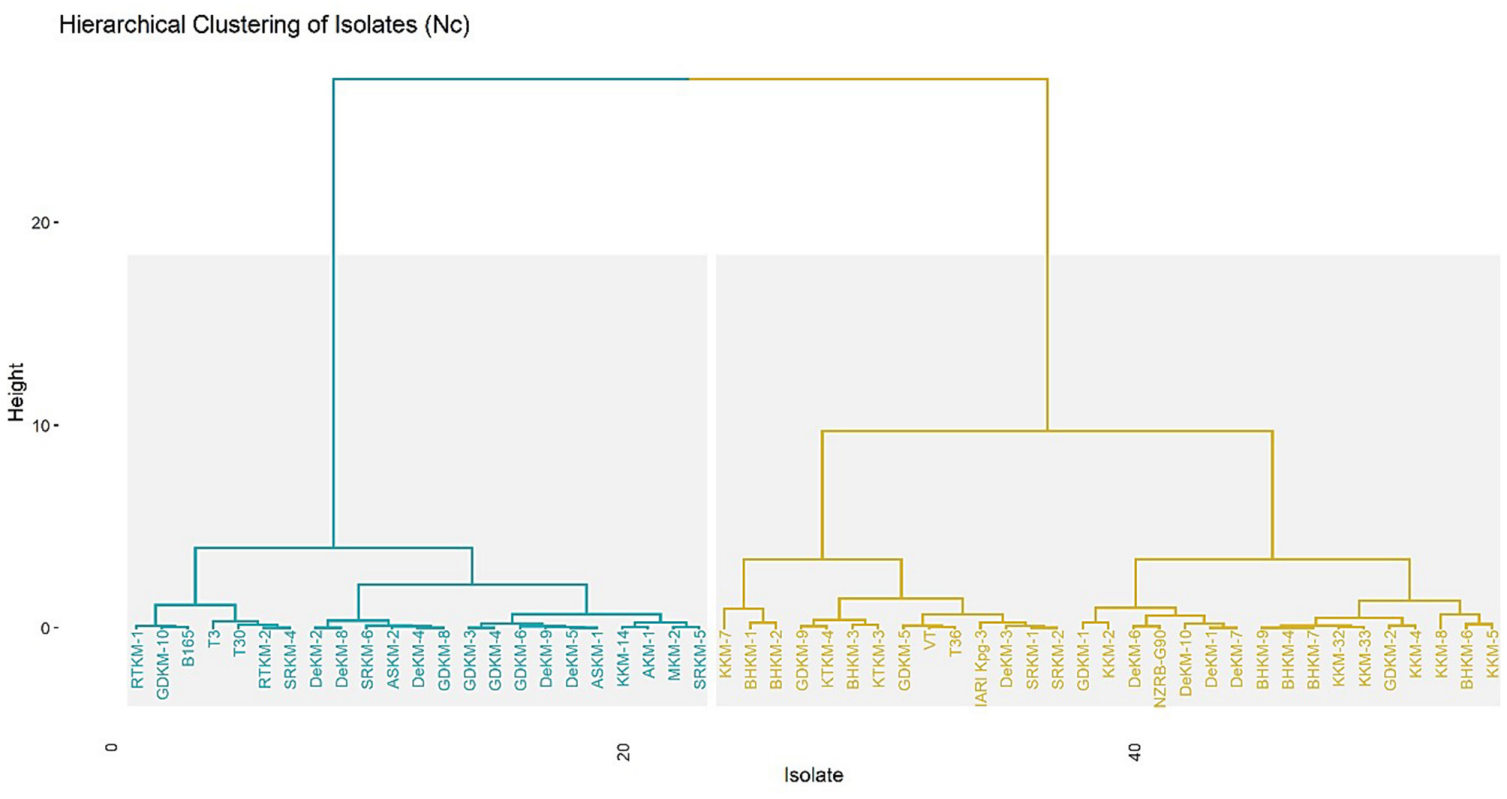
Hierarchical clustering of virus isolates based on ENc values. The *x-axi*s represents individual isolates, while the *y-axis* represents the clustering height. The clustering was performed to explore genetic variation and codon usage patterns across samples from different regions.

A hierarchical clustering dendrogram of virus isolates based on their ENc, highlighting codon usage bias across the isolates. The analysis reveals two distinct clusters: one representing the T30-type and the other the VT-type (Figure 5). The T30-type cluster includes isolates such as T30, RTKM-1, RTKM-2, SRKM-4, and GDKM-10 (20 isolates), which exhibit similar codon usage patterns. Since the T30 strain is internationally recognized as a mild strain, the isolates grouped within this cluster can be considered as putative mild strains, possibly sharing similar biological behavior and host adaptation characteristics. In contrast, the VT-type cluster contains isolates like VT, T36, IARI_Kpg3, and KKM-3 (27 isolates), which are known to be associated with severe infections, indicating higher codon usage divergence and potentially greater virulence.

#### 3.6.1 Codon Adaptation Index (CAI)

**Figure 6:**
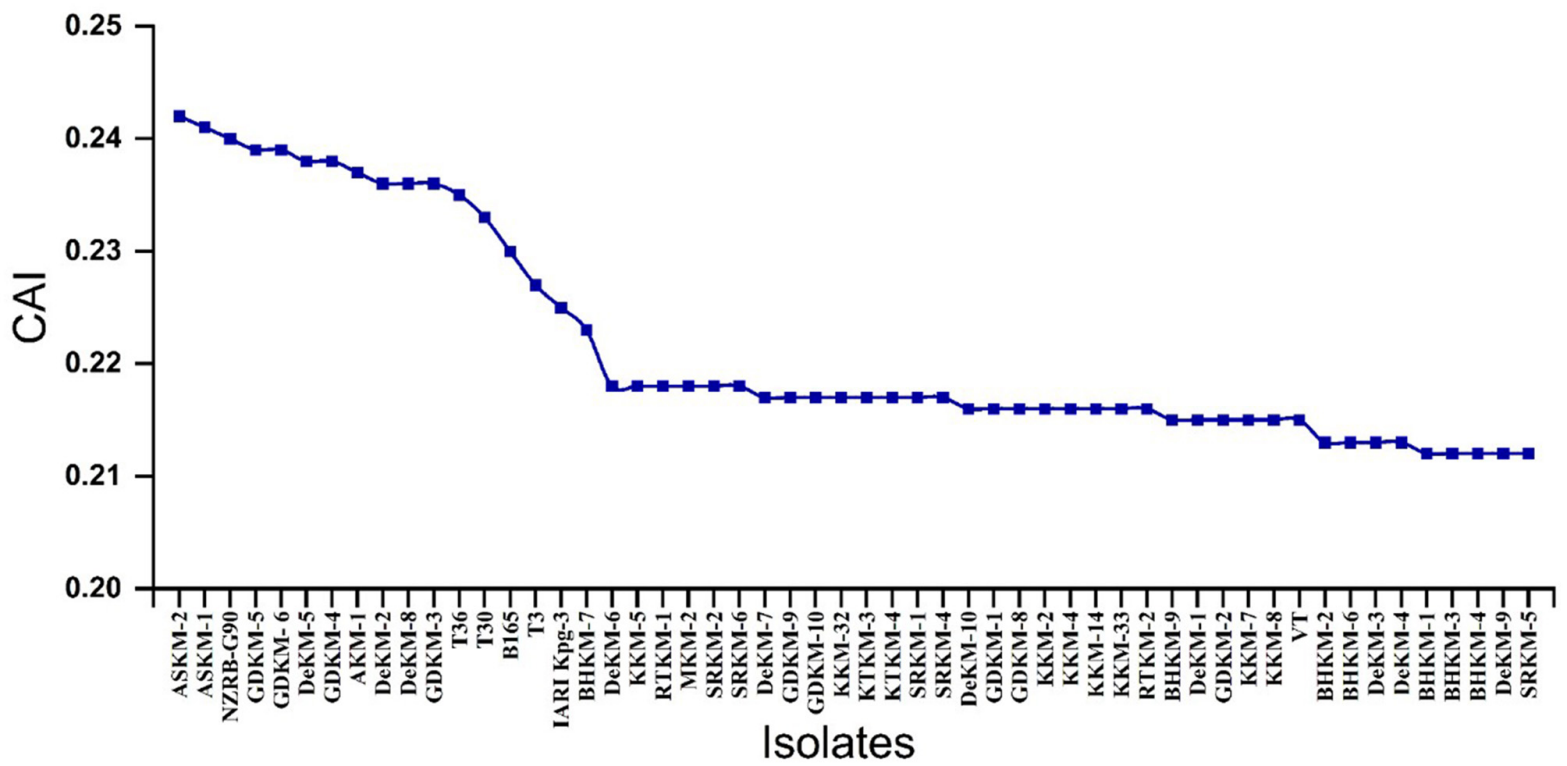
Codon Adaptation Index (CAI) values of different virus isolates. The x-axis represents the different CTV isolates, while the y-axis shows their corresponding CAI values. CAI reflects how well the codon usage of each viral isolate is adapted to the codon preference of the citrus host.

The CAI values ranged from approximately 0.21 to 0.24, indicating variability in codon usage preference (Supplementary Table 5). Notably, putative mild isolates as identified through hierarchical clustering with the T30-type strain exhibited relatively higher CAI values compared to the severe isolates. This suggests that mild isolates may be better adapted to the host's codon usage, potentially allowing more efficient and stable protein expression. In contrast, the lower CAI values observed in the VT-type isolates may reflect a codon usage pattern less optimized for host translation, possibly contributing to altered viral protein synthesis dynamics and increased pathogenicity (Figure 6). This indicates that the mild strain has better adaptation to its host, likely achieved through the selection and incorporation of optimal codons generated during virus replication.

#### 3.6.2 Relative Codon Usage Bias (RCUB) and Principal Component Analysis (PCA) of mild and severe strains of CTV concerning the host

Relative codon usage patterns were analyzed for several amino acids encoded by multiple synonymous codons to evaluate shifts in codon preference across the Cr_Host, DeKM_5 (putative mild strain), and IARI_Kpg_3 (severe strain) samples. For leucine (Leu), encoded by six synonymous codons (UUA, UUG, CUU, CUC, CUA, CUG), the IARI_Kpg_3 strain exhibited a notable increase in the usage of the CUA and CUG codons, while Cr_Host and DeKM_5 displayed a more balanced distribution among all six codons. In the case of valine (Val), encoded by GUU, GUC, GUA, and GUG, IARI_Kpg_3 showed a distinct preference for GUG, diverging from the relatively even usage observed in Cr_Host and DeKM_5. Proline (Pro), encoded by CCU, CCC, CCA, and CCG, showed a sharp elevation in CCG usage in IARI_Kpg_3, whereas CCC remained dominant in both Cr_Host and DeKM_5. Similarly, for serine (Ser), encoded by UCU, UCC, UCA, UCG, AGU, and AGC, IARI_Kpg_3 favored UCC and AGC, indicating a codon usage shift not present in the other two samples.

Alanine (Ala) codons (GCU, GCC, GCA, GCG) revealed further distinctions; Cr_Host and DeKM_5 preferred GCG, while IARI_Kpg_3 predominantly utilized GCC. For arginine (Arg), encoded by CGU, CGC, CGA, CGG, AGA, and AGG, IARI_Kpg_3 exhibited increased use of CGC and AGG, while the host and DeKM_5 maintained more distributed usage. Glutamic acid (Glu), encoded by GAA and GAG, also showed a skew, with IARI_Kpg_3 favoring GAG over GAA. Glycine (Gly), represented by GGU, GGC, GGA, and GGG, was markedly biased toward GGG in the IARI_Kpg_3 strain, in contrast to a more equal representation in the host and mild strain. These codon-specific preferences suggest that IARI_Kpg_3 may be undergoing translational optimization distinct from the host and mild strain, potentially reflecting evolutionary pressure for increased replication efficiency or altered virulence.

**Figure 7:**
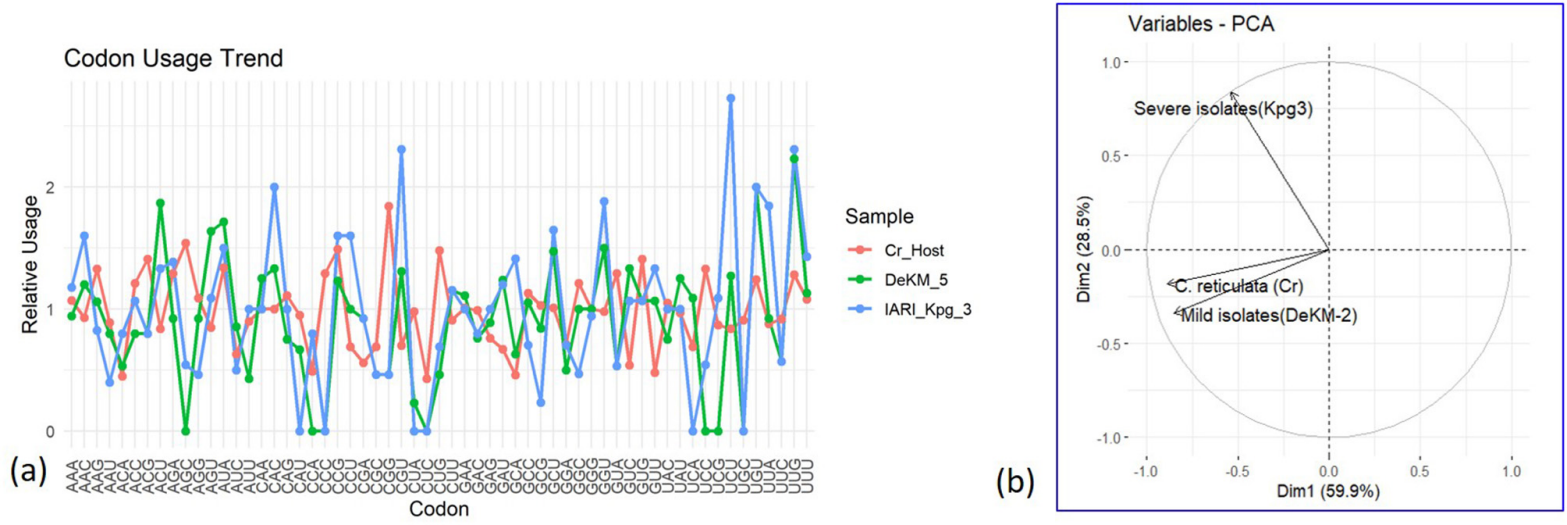
Comparative Analysis of Codon Usage Bias and Principal Component Distribution between Mild and Severe CTV Isolates and Citrus Host. **(a)**: Relative codon usage bias (RCUB); **(b**): Principal Component Analysis (PCA) of mild and severe strains of CTV concerning the Host.

The codon usage patterns of DeKM-5 (putative mild strain) closely follow that of the host (Cr. Host) for most codons, indicating a higher degree of codon adaptation. This similarity suggests that DeKM-5 may rely on optimized translation efficiency by aligning its codon preferences with the host's tRNA pool, contributing to reduced pathogenicity and more stable replication. In contrast, the IARI Kpg3 exhibits a more divergent codon usage profile, particularly at several codon positions where the relative usage differs sharply from both the host and DeKM-5. This deviation may reflect a lower level of adaptation to host codon usage and potentially result in inefficient translation or host stress, which could contribute to its higher virulence (Figure 7a). Overall, this analysis suggests that mild strains tend to have codon usage patterns more similar to the host, while severe strains show greater deviation, supporting the hypothesis that codon usage bias plays a role in viral fitness and pathogenicity.

#### 3.6.3 Principal component analysis (PCA) of codon usage bias reveals distinct clustering between CTV strains and host

The PCA biplot revealed distinct clustering patterns. The *C. reticulata* host and the mild strain DeKM-2 grouped closely in the lower left quadrant, suggesting similar codon usage profiles. The first two principal components (Dim1 and Dim2) accounted for a cumulative 88.4% of the total variance, with Dim1 explaining 59.9% and Dim2 explaining 28.5%.In contrast, the severe strain Kpg3 was separated in the upper left quadrant along Dim2, indicating substantial divergence in codon usage bias compared to both the host and the mild strain (Figure 7b).This separation suggests that the severe strain Kpg3 has undergone adaptive evolution, resulting in a codon usage pattern that differs markedly from that of the host and the mild strain. The proximity of the mild strain to the host implies possible host adaptation or co-evolution, potentially contributing to its attenuated pathogenicity. The distinct positioning of the severe isolate highlights its codon optimisation strategy, which may support enhanced translational efficiency or evasion of host defenses, thereby contributing to increased virulence. Overall, the PCA analysis supports the hypothesis that codon usage bias plays a critical role in the evolution and pathogenic differentiation of CTV strains.

#### 3.6.3 Nucleotide composition of putative mild, severe and host

**Figure 8:**
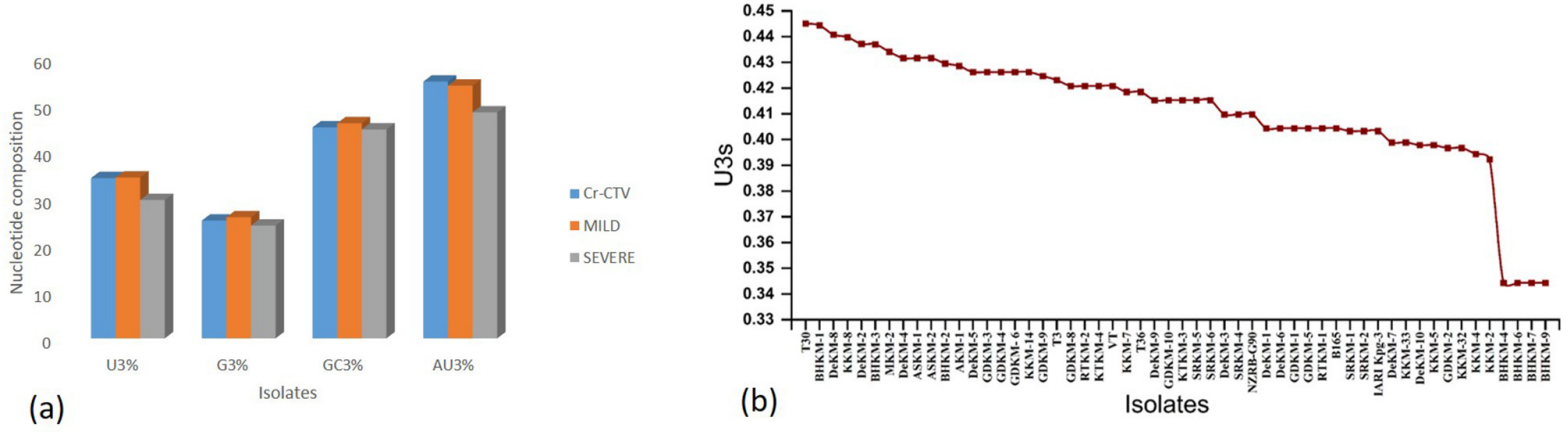
Nucleotide composition and U3s codon bias in CTV isolates. **(a):** Bar plot showing comparative nucleotide composition (U3%, G3%, GC3%, and AU3%) among the overall CTV population, mild isolates, and severe isolates. **(b)** Line graph illustrating U3s (uracil at the third codon position of synonymous codons) across individual CTV isolates.

In the present study, nucleotide composition analysis revealed a preference for U-ending codons in the CTV CP gene. Interestingly, the putative mild isolates and the host exhibited higher Uracil content at the third codon position than the severe isolates. This higher Uracil preference may provide the mild isolates with an advantage for efficient replication in the host cell by reducing competition with the host for the cellular machinery (Figure 8).

Based on the combined analysis of ENC values, CAI values, and the prevalence of Uracil at the third codon position, we have identified nine promising putative mild strains: DeKM-2, ASKM-1, ASKM-2, AKM-1, GDKM-3, GDKM-4, DeKM-6, DeKM-8, and DeKM-5. These isolates exhibited higher CAI values, suggesting better adaptation to the host's codon usage pattern, along with lower CUB. Additionally, the increased presence of U-ending codons in these isolates further supports their efficient utilization of the host's translational machinery.

#### 3.6.4 Quantitative RT-PCR (qpcr) assay for the viral load in leaf tissues of CTV-infected plants with mild and severe strains

**Figure 9:**
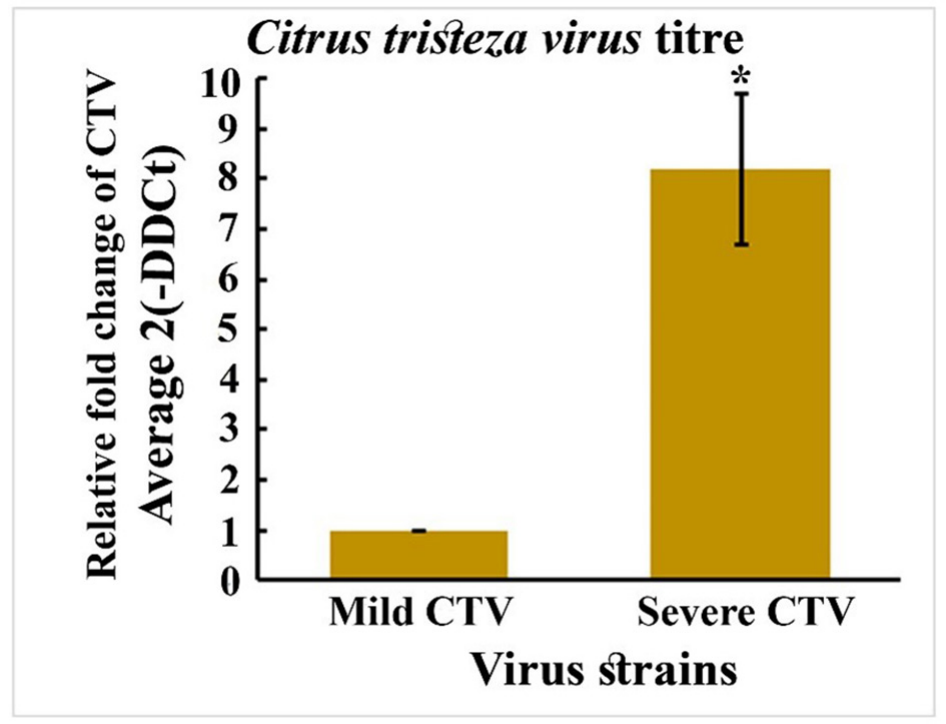
Quantitative RT-PCR (qPCR) assay for the viral load in leaf tissues CTV infected with Mild and severe strains.

The relative viral titer, expressed as fold change using the 2^Λ−^ΔΔCt method, was significantly lower in mild strain-infected plants than in Severe strain-infected plants. Using mild strain as the baseline (1-fold), the viral load in severe infected plants showed 9-fold increase. In contrast, the mild strain showed minimal accumulation, suggesting limited replication and systemic movement within the host. Statistical analysis confirmed that the observed increase in titer for the severe strain was highly significant (*p* < 0.05), as indicated by the error bar and asterisk marker (Figure 9). The elevated viral load in severe isolates correlates with their higher pathogenicity and likely reflects more efficient replication, cell-to-cell movement, and suppression of host defenses.

### 3.7 Putative mild strains were grafted on different rootstocks and further challenged inoculate with a severe strain of CTV

To validate the mild nature of putative cross-protecting CTV strains, five putative mild isolates **(**DeKM-2, ASKM-1, ASKM-2, DeKM-8, and DeKM-5) were biologically indexed through graft inoculation on two standardized indicator hosts *Citrus aurantifolia* (Kagzi lime) and *Citrus jambhiri* (Rough lemon). Each strain was grafted with three biological replicates under controlled greenhouse conditions.

Observations recorded after six months revealed that on the indicator hosts (kagzi lime), symptoms such as very mild vein clearing, mild stunting were observed. Whereas, plants grafted on Rough lemon rootstock showed no symptoms.

The grafted plants were subsequently challenge-inoculated with the severe strain (Kpg3) through grafting; however, no typical symptoms, such as vein clearing, were observed even after three months, thereby confirming the mild pathogenic nature and potential cross-protective ability of the tested isolates. These findings provide phenotypic confirmation that the five CTV isolates under investigation are indeed mild strains (Table 2). Their limited symptom expression on highly susceptible indicator hosts supports their potential utility in mild strain cross-protection strategies for citrus orchards threatened by severe CTV strains and control (Severe strain-inoculated) seedlings exhibited typical symptoms like vein clearing and vein flecking.

**Table 2:** Biological indexing of Putative mild strain on Kagzi lime and Rough lemon.

**Isolates**	**Symptoms appeared in the inoculated plant(Number of plants producing symptoms/Number of plants inoculated)**	**Kagzi lime**	**Rough lemon**	**ELISA**	**PCR**
DeKM-2	5/6	Mild Vcl	Healthy	+	+
ASKM-1	4/5	Mild Vcl	Healthy	+	+
ASKM-2	6/6	Mild St, PG	Healthy	+	+
DeKM-8	5/6	Mild Vcl, PG	Healthy	+	+
DeKM-5	5/6	Mild Vcl	Healthy	+	+

### 3.8 Sequence identity matrix and phylogenetic analysis of mild and severe strain of CTV

Pairwise sequence analysis based on the CP gene of the present isolate 47, along with previously reported Indian and international CTV isolates, revealed that mild and severe strains share 91–92% nucleotide identity, while mild strains among themselves share a higher identity of 97–99%. The mild isolates DeKM-2, ASKM-1, ASKM-2, AKM-1, GDKM-3, GDKM-4, DeKM-6, DeKM-8, and DeKM-5 showed close genetic similarity to the severe strain VT, sharing up to 98% nucleotide identity. These findings suggest that mild strains are genetically similar to severe strains, and according to the cross-protection principle, they are suitable candidates for managing severe CTV strains. Therefore, the isolates DeKM-2, ASKM-1, ASKM-2, AKM-1, GDKM-3, GDKM-4, DeKM-6, DeKM-8, and DeKM-5 can be considered reliable mild strains for cross-protection strategies.

**Figure 10:**
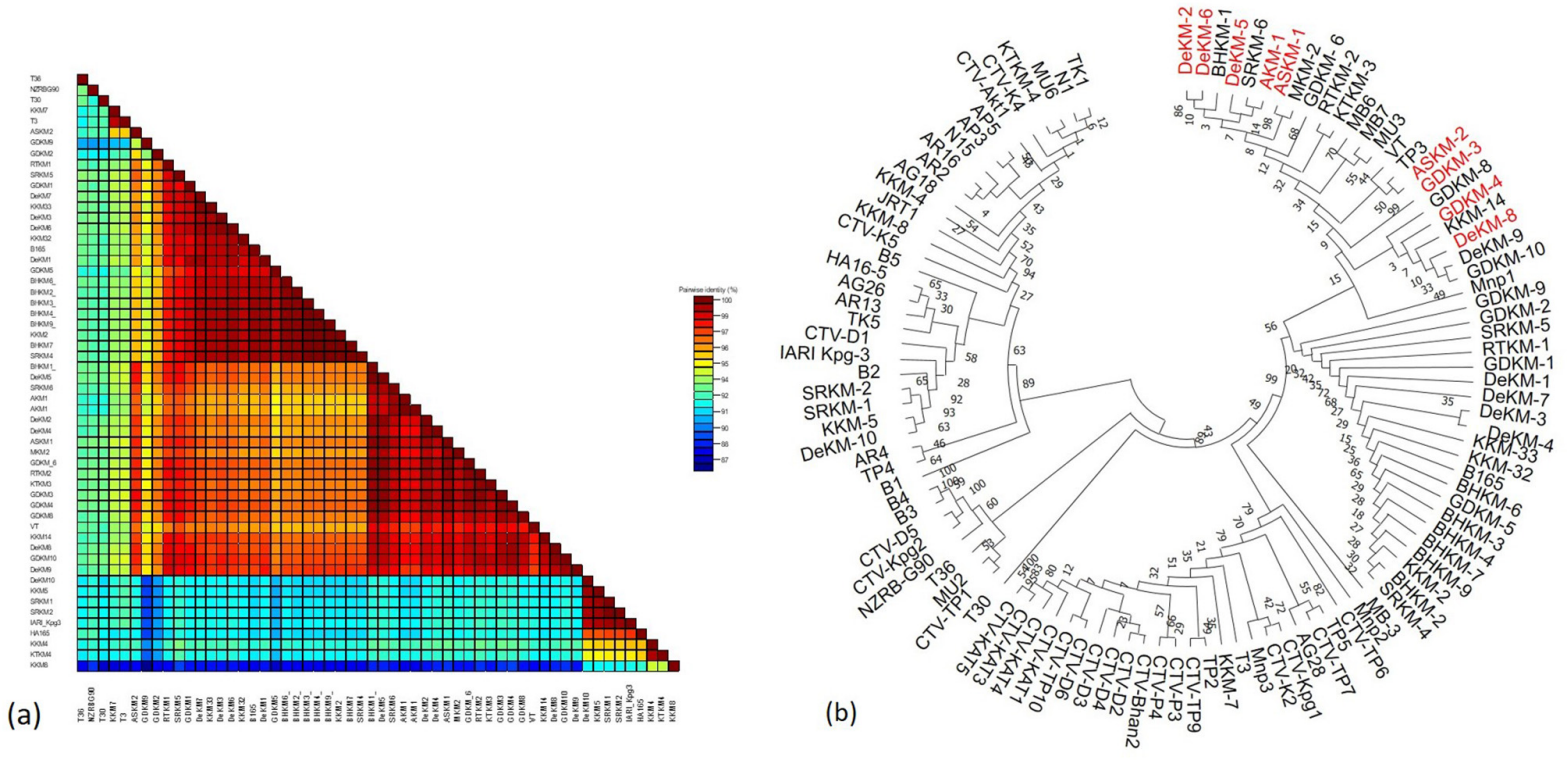
**(a**) Color-coded pairwise nucleotide identity percent matrix of CTV isolates based on the CP gene (87 to 100%); (**b**) Neighbor-joining phylogenetic analysis for nucleotide sequences of the CP gene of CTV isolates using MEGA 11.

Neighbor-joining phylogenetic analysis of the nucleotide sequences of the CP gene of CTV isolates revealed that the mild strains form a distinct clade, clustering closely together and indicating a high degree of genetic similarity and a possible shared evolutionary origin. These mild isolates exhibit less genetic divergence than other CTV strains, suggesting low virulence and potential utility in cross-protection against severe strains. The distinct clustering of these mild strains, separate from more virulent or divergent isolates, supports the hypothesis of strain-specific pathogenicity and evolutionary divergence. Interestingly, the clade of mild strains also includes the severe strain VT, highlighting their genetic relatedness (Figure 10).

### 3.9 Biochemical analysis

#### 3.9.1 Total sugar content between mild and severe strain

**Figure 11:**
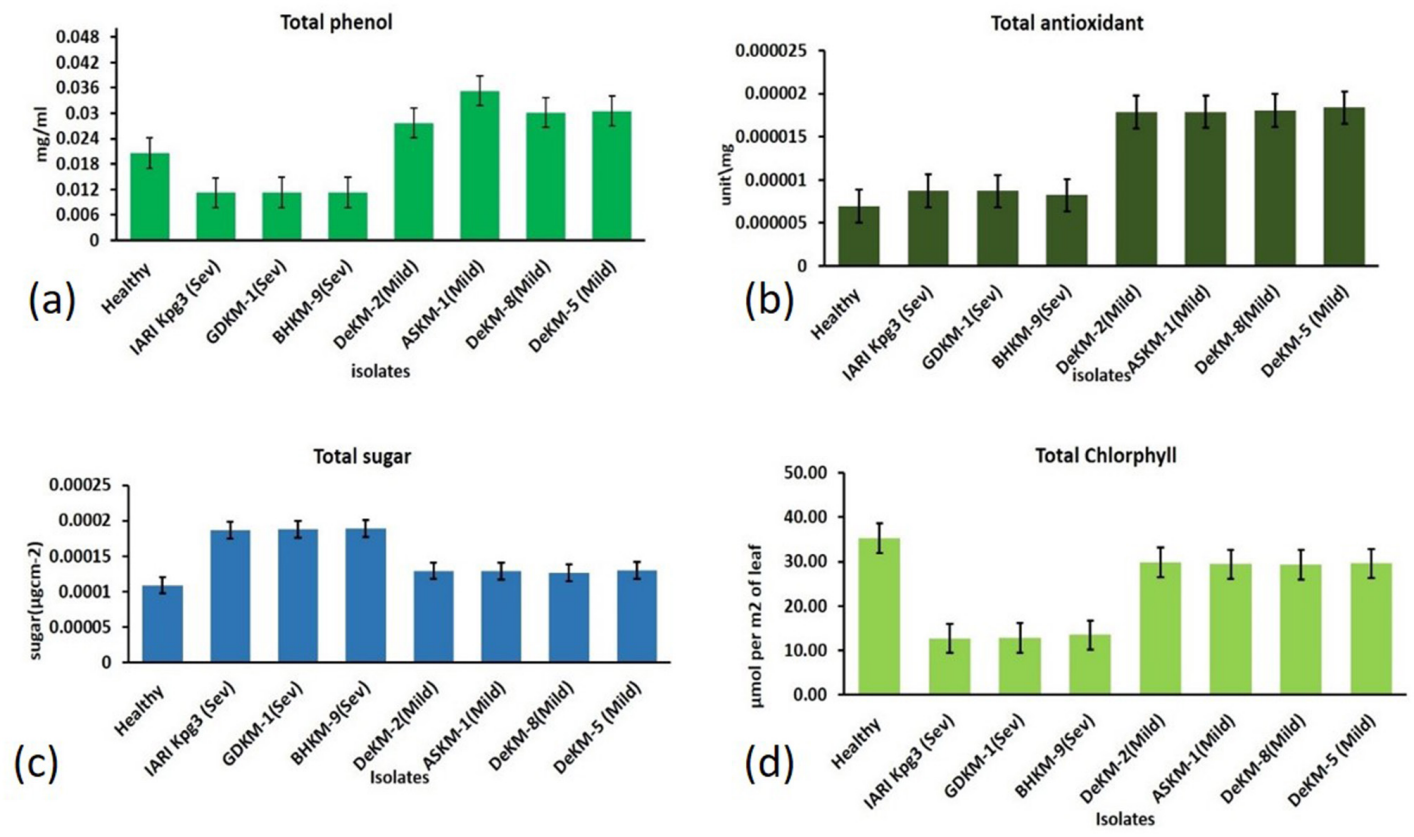
Biochemical Responses in Citrus Leaves Infected with Mild and Severe CTV Isolates Compared to Healthy Controls: **(a**) Total phenol; **(b**) Total antioxidant; **(c**) Total sugar; (**d**) Total chlorophyll.

Understanding how plant pathogenic viruses affect sugar metabolism in infected hosts is essential for assessing their economic impact. Plants infected with the severe strain exhibited the highest sugar content (0.219 mg/100g), possibly due to stress-induced metabolic changes or virus-induced alterations in carbohydrate metabolism. This was followed by plants infected with the mild strain (0.151 mg/100g), while healthy plants had the lowest sugar content (0.128 mg/100g) (Figure 11a).

#### 3.9.2 Total antioxidants estimation

The total antioxidant content was highest in plants infected with the mild strain (0.0180 mg/100g), followed by those infected with the severe strain (0.0085 mg/100g), and lowest in healthy plants (0.0069 mg/100g) (Figure 11b).

#### 3.9.3 Total phenol content

Total phenol content was highest in plants infected with the mild strain (35.95 mg/100g), followed by healthy plants (24.22 mg/100g), and lowest in plants infected with the severe strain (13.00 mg/100g) (Figure 11c).

#### 3.9.4 Total chlorophyll concentration

The Chlorophyll Concentration Index (CCI), measured using the MC-100 Chlorophyll Concentration Meter (Apogee Instruments), revealed that chlorophyll content was highest in healthy plants (35.23 μmol m^−2^), followed by plants infected with the mild strain (29.5 μmol m^−2^), and lowest in those infected with the severe strain (13.00 μmol m^−2^) (Figure 11d).

## 4 Discussion

In the absence of natural resistance to CTV and the infeasibility of rapid replanting with tolerant or resistant rootstocks, Mild Strain Cross-Protection (MSCP) emerges as a biologically grounded and sustainable strategy for disease management in citrus. MSCP is a phenomenon wherein a prior infection with a mild strain of the same virus can prevent symptom development upon subsequent infection with a virulent strain (Lee et al., 1987). This method is particularly appealing as a non-transgenic, field-applicable solution that capitalizes on naturally occurring virus-virus interactions and offers long-term disease mitigation. Its effectiveness hinges on strain specificity and genetic compatibility, underlining the necessity for rigorous identification and validation of mild strains suitable for cross-protection. Although previous research has addressed aspects of host-CTV interaction and viral adaptation (Cheng et al., 2012; Biswas et al., 2019), it has not attempted to identify specific mild strains or conduct detailed comparisons between mild and severe CTV strains in terms of codon usage and host adaptation mechanisms representing a major gap in the effective implementation of MSCP.

This study addresses a significant gap in CTV research by providing a comprehensive molecular, evolutionary, and biochemical distinction between mild and severe strains affecting *Citrus reticulata*. We analyzed forty seven field-derived isolates maintained under controlled conditions at ICAR-IARI in New Delhi. Our integrated approach included codon usage bias analysis (CAI, RCUB, PCA), quantification of viral titer via qRT-PCR, biological indexing, and detailed biochemical profiling to better understand the complexities of CTV-host interactions. Our primary goal was to identify candidate mild strains with the potential for cross-protection, and we have successfully achieved this through rigorous comparative analyses. This research enhances our understanding of host adaptation and viral behavior while providing actionable insights for implementing MSCP as a sustainable, non-transgenic approach to CTV management. By identifying evolutionary and functional biochemical markers that differentiate protective mild strains from virulent ones, this work establishes a solid scientific basis for developing future field-ready MSCP strategies tailored to Indian citrus production systems.

A promising approach to exploring these differences is the analysis of CUB, which reflects the evolutionary relationship between a virus and its host's translational machinery. CUB is influenced by mutational pressure and natural selection (Belalov and Lukashev, 2013) and can serve as a molecular indicator of host adaptation. In our study, we analyzed isolates from 117 CTV and found ENc values ranging from 49.29 to 59.2, with a mean of 53.96 ± 0.22. The GC3 values ranged from 0.31 to 0.42. This distribution indicates that both evolutionary forces are at work. However, neutrality plot analysis showed a narrow GC3 range and no significant correlation with GC12, suggesting that natural selection predominates over mutational bias. These findings are consistent with earlier reports by Sharp and Li (1986), which highlight the selective constraints on synonymous codon usage in RNA viruses. Further insights were gained from correlation analysis, which revealed significant positive correlations between ENc and both C3 and A3, along with a significant negative correlation between ENc and U3. These results imply that increased uracil frequency at the third codon position is associated with stronger codon bias, consistent with previous studies highlighting the influence of nucleotide composition on CUB in both RNA and DNA viruses (Nakamura et al., 2000; Cheng et al., 2012). The generally high ENc values observed in our dataset support the idea of low codon usage bias in CTV, a characteristic often reported in other RNA viruses such as Ebola (Cristina et al., 2015), SARS-CoV-2 (Kandeel et al., 2020), and mycoviruses (Wang et al., 2022). This low CUB may confer greater adaptability across diverse citrus genotypes by allowing the virus to utilize host codon pools more flexibly.It is expected that a greater similarity in codon usage patterns will enhance viral replication. The degree of codon usage compatibility between viruses and their hosts has been proposed to influence viral survival, fitness, and the ability to evade the host immune response (Burns et al., 2006; Costafreda et al., 2014; Mueller et al., 2006).

Interestingly, clustering analysis based on ENc values revealed that T30-type isolates, which are globally recognized as mild strains, formed a distinct group. Out of forty seven present isolates, twenty clustered with T30-types and were designated as putative mild strains. These mild isolates exhibited lower codon bias, potentially reflecting a more advantageous relationship with host translational machinery and reduced replication-driven stress on host cells (Tian et al., 2018; Wang et al., 2020; Tsai et al., 2007). Nucleotide composition analysis further supported this observation, as mild strains displayed higher uracil frequency at the third codon position similar to the host, compared to severe strains, indicating earlier findings of U-ending codon preference in citrus hosts (Ahmad et al., 2013).

RSCU analysis revealed that the codon usage of mild strains was more aligned with that of citrus hosts, while the severe strain (Kpg3) diverged significantly, indicating greater bias. PCA biplot analysis reinforced this distinction, clustering mild strains and the host together while separating the severe strain into a distinct cluster. This divergence may reflect evolution in the severe strain. Further quantitative RT-PCR analysis showed that the severe strain had approximately ninefold higher viral titer than the mild strain, indicating the severe strain is replicating at a higher rate than the mild strain. While such high replication levels may enhance short-term infectivity (Elena and Sanjuán, 2005), they could compromise long-term viral fitness due to error-prone replication by the RNA-dependent RNA polymerase (RdRP). In contrast, the mild strains likely maintain a balance between efficient replication and long-term persistence through higher fidelity replication, thereby supporting compatibility with host physiological systems (Jenkins and Holmes, 2003).

Five putative mild strains DeKM-2, ASKM-1, ASKM-2, DeKM-8, and DeKM-5 were identified based on a combination of codon usage metrics, including high CAI, ENc values, and a prevalence of U-ending codons. These features suggest optimized usage of host translational machinery, supporting their candidacy for cross-protection. The cross-protective potential of these putative mild strains was validated through biological indexing on *Citrus aurantifolia* (indicator host*)* and *Citrus jambhiri (*tolerant host*)*. After seven months, the mild strains induced only mild symptoms such as slight vein clearing and flecking on Kagzi lime, while no symptoms were observed on Rough lemon. The presence of the virus in both hosts was confirmed using ELISA and RT-PCR. Crucially, plants pre-inoculated with these mild strains showed no severe symptoms when challenge-inoculated with the virulent Kpg3 strain even after 3 months, confirming effective cross-protection. These observations are consistent with prior studies demonstrating superinfection exclusion in MSCP systems (Moreno et al., 1993; Lee and Keremane, 2013).

For a strain to be considered as mild, it should share a sequence similarity to the severe strain, and in our study, the Phylogenetic analysis confirmed that the nine mild strains share 98% nucleotide identity with the severe VT strain and grouped within a single clade. This genetic proximity is critical for the phenomenon of superinfection exclusion, which is more effective among closely related viral strains (Folimonova et al., 2010; Folimonova, 2020). The observed genetic similarity, combined with phenotypic attenuation, supports the idea that the mild strains originated from a common lineage but have diverged in virulence, potentially through point mutations affecting viral protein-host interactions.

Furthermore, a comparative biochemical analysis was conducted to assess host physiological responses. The highest sugar content was observed in plants infected with the severe strain, potentially reflecting disrupted phloem function or altered source-sink dynamics under stress (Sheen et al., 1999; Smeekens, 2000; Addy et al., 2017). In contrast, antioxidant content peaked in mild strain-infected plants, suggesting a robust, non-lethal defense response (Singh et al., 2022). Elevated phenolic content in these plants also points to enhanced antiviral defense, consistent with earlier work highlighting the role of phenolics in systemic acquired resistance (Link and Walker, 1933; Rashad et al., 2020). Chlorophyll content was highest in healthy plants and lowest in those infected with the severe strain, indicating photosynthetic disruption and oxidative stress (Masaló and Baradad, 2020). These biochemical markers serve as important indicators of the physiological toll exacted by severe strains and reinforce the relative compatibility of mild strains with the host plant. The integration of codon usage profiling, viral load quantification, phylogenetics, and host biochemical responses provides a robust framework for differentiating CTV strains based on their adaptation and pathogenic potential. The identification and validation of mild strains in this study not only advance our understanding of CTV evolution but also hold practical implications for developing effective cross-protection strategies in regions severely affected by this virus, such as Northeast India.

## 5 Conclusion

This study highlights the potential of mild CTV strains as effective biological agents for cross-protection in Khasi mandarin. By integrating codon usage bias analysis, host adaptation indices, phylogenetic, biochemical assays, and biological indexing, we identified five putative mild strains DeKM-2, ASKM-1, ASKM-2, DeKM-8, and DeKM-5 that exhibit low pathogenicity, strong codon adaptation to the citrus host, and high genetic similarity to virulent strains. These strains displayed reduced codon usage bias and elevated CAI values, suggesting optimized translational compatibility. Biological indexing confirmed their mild nature, while biochemical assays revealed moderate physiological changes compared to severe and healthy controls, indicating minimal disruption of host metabolism. In the absence of natural resistance and the impracticality of large-scale replanting, these well-characterized mild strains offer a sustainable, region-specific, and non-transgenic approach to CTV management. Future work should focus on long-term field validation across diverse agro-climatic zones to confirm their durability and efficacy in real-world orchard settings.

## Data Availability

The original contributions presented in the study are included in the article/Supplementary material, further inquiries can be directed to the corresponding author.

## References

[B1] Adams M. J. Antoniw J. F. (2003). Codon usage bias amongst plant viruses. Arch. Virol. 149, 113–135. 10.1007/s00705-003-0186-6 14689279

[B2] Addy H. S. Nurmalasari W. ahyudi A. H. S. Sholeh A. Anugrah C. Iriyanto F. E. S. Darmanto W.. (2017). Detection and response of sugarcane against the infection of *Sugarcane mosaic virus* (SCMV) in Indonesia. Agronomy 7:50. 10.3390/agronomy7030050

[B3] Ahlawat Y. S. Pant R. P. (2003). Major virus and virus-like diseases of citrus in India: their diagnosis and management. Annu. Rev. Plant Pathol. 2, 447–474.

[B4] Ahmad T. Sablok G. Tatarinova T. V. Xu Q. Deng X. X. Guo W. W. (2013). Evaluation of codon biology in *Citrus* and *Poncirus trifoliata* based on genomic features and frame-corrected expressed sequence tags. DNA Res. 20, 135–150. 10.1093/dnares/dss039 23315666 PMC3628444

[B5] Albiach-Marti M. R. (2013). “The complex genetics of *Citrus tristeza virus*,” in Current Issues in Molecular Virology – Viral Genetics and Biotechnological Applications. Books on Demand, ed. V. Romanowski (Norderstedt: Germany), 1–26.

[B6] Ashfaq M. Iqbal S. Mukhtar T. Shah H. (2014). Screening for resistance to *Cucumber mosaic cucumovirus* in chilli pepper. J. Anim. Plant Sci. 24, 791–795.

[B7] Bar-Joseph M. Marcus R. Lee R. F. (1989). The continuous challenge of *Citrus tristeza virus* control. Annu. Rev. Phytopathol. 27, 291–316. 10.1146/annurev.py.27.090189.001451

[B8] Bar-Joseph M. Marcus R. Lee R. F. (2002). The continuous challenge of *Citrus tristeza virus* control. Annu. Rev. Phytopathol. 27, 291–316. 10.1146/annurev.py.27.090189.001451

[B9] Belalov I. S. Lukashev A. N. (2013). Causes and implications of codon usage bias in RNA viruses. PLoS ONE 8:e56642. 10.1371/journal.pone.0056642 23451064 PMC3581513

[B10] Benzie I. F. Strain J. J. (1999). Ferric reducing/antioxidant power assay: direct measure of total antioxidant activity of biological fluids and modified version for simultaneous measurement of total antioxidant power and ascorbic acid concentration. Methods Enzymol. 299, 15–27. 10.1016/S0076-6879(99)99005-5 9916193

[B11] Biswas K. K. (2008). Molecular methods of diagnosis of *Citrus tristeza virus* in mandarin (*Citrus reticulata*) orchard in Darjeeling hills of West Bengal. Indian J. Virol. 19, 30–36.

[B12] Biswas K. K. (2010). Molecular characterization of *Citrus tristeza virus* isolates from the Northeastern Himalayan region of India. Arch. Virol. 155, 959–963. 10.1007/s00705-010-0602-7 20437063

[B13] Biswas K. K. Palchoudhury S. Chakraborty P. Bhattacharyya U. K. Ghosh D. K. Debnath P.. (2019). Codon usage bias analysis of *Citrus tristeza virus*: higher codon adaptation to *Citrus reticulata* host. Viruses 11:331. 10.3390/v11040331 30965565 PMC6521185

[B14] Biswas K. K. Tarafdar A. Sharma S. K. (2012). Complete genome of mandarin decline *Citrus tristeza virus* of Northeastern Himalayan hill region of India: comparative analyses determine recombinant. Arch. Virol. 157, 579–583. 10.1007/s00705-011-1165-y 22160128

[B15] Burns C. C. Shaw J. Campagnoli R. Jorba J. Vincent A. Quay J.. (2006). Modulation of poliovirus replicative fitness in HeLa cells by deoptimization of synonymous codon usage in the capsid region. J. Virol. 80, 3259–3272. 10.1128/JVI.80.7.3259-3272.2006 16537593 PMC1440415

[B16] Chakraborty P. Das S. Saha B. Sarkar P. Karmakar A. Saha A.. (2015). Phylogeny and synonymous codon usage pattern of *Papaya ringspot virus* coat protein gene in the sub-Himalayan region of north-east India. Can. J. Microbiol. 61, 555–564. 10.1139/cjm-2015-0172 26114545

[B17] Chaney J. L. Clark P. L. (2015). Roles for synonymous codon usage in protein biogenesis. Annu. Rev. Biophys. 44, 143–166. 10.1146/annurev-biophys-060414-034333 25747594

[B18] Cheng C. Yang J. Yan H. Bei X. Zhang Y. Lu Z.. (2015). Expressing p20 hairpin RNA of *Citrus tristeza virus* confers *Citrus aurantium* with tolerance/resistance against stem pitting and seedling yellow CTV strains. J. Integr. Agric. 14, 1767–1777. 10.1016/S2095-3119(14)60937-0

[B19] Cheng X. F. Wu X. Y. Wang H. Z. Sun Y. Q. Qian Y. S. Luo L. (2012). High codon adaptation in *Citrus tristeza virus* to its citrus host. Virol. J. 9:113. 10.1186/1743-422X-9-113 22698086 PMC3416656

[B20] Costa A. S. Müller G. W. (1980). Tristeza control by cross-protection: a U.S.-Brazil cooperative success. Plant Dis. 64, 538–541. 10.1094/PD-64-538

[B21] Costafreda M. I. Perez-Rodriguez F. J. D'Andrea L. Guix S. Ribes E. Bosch A.. (2014). Hepatitis A virus adaptation to cellular shutoff is driven by dynamic adjustments of codon usage and results in the selection of populations with altered capsids. J. Virol. 88, 5029–5041. 10.1128/JVI.00087-14 24554668 PMC3993836

[B22] Cristina J. Moreno P. Moratorio G. Musto H. (2015). Genome-wide analysis of codon usage bias in Ebolavirus. Virus Res. 196, 87–93. 10.1016/j.virusres.2014.11.005 25445348

[B23] Elena S. F. Sanjuán R. (2005). Adaptive value of high mutation rates of RNA viruses: Separating causes from consequences. J. Virol. 79, 11555–11558. 10.1128/JVI.79.18.11555-11558.2005 16140732 PMC1212614

[B24] EPPO (2023). PM 7/31 (2) *Citrus tristeza virus*. EPPO Bull. 53, 42–61. 10.1111/epp.12908

[B25] FAO (2023). Available online at: http://www.fao.org/faostat/en/#data

[B26] Febres V. J. Ashoulin L. Mawassi M. Frank A. Bar-Joseph M. Manjunath K. L.. (1996). The p27 protein is present at one end of *Citrus tristeza virus* particles. Phytopathology 86, 1331–1335.

[B27] Foley J. A. Ramankutty N. Brauman K. A. Cassidy E. S. Gerber J. S. Johnston M.. (2011). Solutions for a cultivated planet. Nature 478, 337–342. 10.1038/nature10452 21993620

[B28] Folimonova S. Y. (2020). *Citrus tristeza virus*: a large RNA virus with complex biology turned into a valuable tool for crop protection. PLoS Pathog. 16:e1008416. 10.1371/journal.ppat.1008416 32353070 PMC7192379

[B29] Folimonova S. Y. Robertson C. J. Shilts T. Folimonov A. S. Hilf M. E. Garnsey S. M.. (2010). Infection with strains of *Citrus tristeza virus* does not exclude superinfection by other strains of the virus. J. Virol. 84, 1314–1325. 10.1128/JVI.02075-09 19923189 PMC2812332

[B30] Forner-Giner M. A. Primo-Millo E. Forner J. B. (2009). Performance of Forner-Alcaide 5 and Forner-Alcaide 13, hybrids of Cleopatra mandarin × *Poncirus trifoliata*, as salinity-tolerant citrus rootstocks. J. Am. Pomol. Soc. 63, 72–80.

[B31] Franzo G. Tucciarone C. M. Cecchinato M. Drigo M. (2017). Canine parvovirus type 2 (CPV-2) and feline panleukopenia virus (FPV) codon bias analysis reveals a progressive adaptation to the new niche after the host jump. Mol. Phylogenet. Evol. 114, 82–92. 10.1016/j.ympev.2017.05.019 28603036

[B32] Fraser L. R. Long K. Cox J. (1968). Stem pitting of grapefruit—field protection by the use of mild virus strains. Proc. Int. Organ. Citrus Virol. 4, 27–31. 10.5070/C529Q2V255

[B33] Gorinstein S. Zachwieja Z. Katrich E. Pawelzik E. Haruenkit R. Trakhtenberg S.. (2004). Comparison of the contents of the main antioxidant compounds and the antioxidant activity of white grapefruit and his new hybrid. LWT - Food Sci. Technol. 37, 337–343. 10.1016/j.lwt.2003.10.005

[B34] Gottwald T. R. Polek M. Riley K. (2002). “History, present incidence, and spatial distribution of *Citrus tristeza virus* in the California Central Valley,” in Proceedings of the Fifteenth Conference of the International Organization of Citrus Virologists, eds. N. Duran-Vila, R. G. Milne, J.V Da Graça (IOCV, CA: Riverside), 83–94. 10.5070/C51561486J

[B35] Guarino S. Abbate L. Mercati F. Fatta Del Bosco S. Motisi A. Arif M. A.. (2021). Citrus varieties with different tolerance grades to *Citrus tristeza virus* show dissimilar volatile terpene profiles. Agronomy 11:1120. 10.3390/agronomy11061120 PMC9788239 36559538

[B36] Harper S. J. (2013). *Citrus tristeza virus*: Evolution of complex and varied genotypic groups. Front. Microbiol. 4, 1–18. 10.3389/fmicb.2013.00093 23630519 PMC3632782

[B37] Hernández J. A. Rubio M. Olmos E. Ros-Barcel,ó A. Martínez-Gómez P. (2004). Oxidative stress induced by long-term *Plum pox virus* infection in peach (*Prunus persica* L. cv GF305). Physiol. Plant 122, 486–495. 10.1111/j.1399-3054.2004.00431.x

[B38] Hilf M. E. Karasev A. V. Pappu H. R. Gumpf D. J. Niblett C. L. Garnsey S. M. (1995). Characterization of *Citrus tristeza virus* subgenomic RNAs in infected tissue. Virology 208, 576–582. 10.1006/viro.1995.1188 7747429

[B39] Jenkins G. M. Holmes E. C. (2003). The extent of codon usage bias in human RNA viruses and its evolutionary origin. Virus Res. 92, 1–7. 10.1016/S0168-1702(02)00309-X 12606071

[B40] Jyoti Y.easrin, S. Kalita I. Tanti B. (2023). Phytochemical screening, proximate composition and antioxidant activities of *Citrus* germplasm of Assam, India. Vegetos 37, 1153-1165. 10.1007/s42535-023-00666-6

[B41] Kandeel M. Ibrahim A. Fayez M. Al-Nazawi M. (2020). From SARS and MERS CoVs to SARS-CoV-2: Moving toward more biased codon usage in viral structural and nonstructural genes. J. Med. Virol. 92, 660–666. 10.1002/jmv.25754 32159237 PMC7228358

[B42] Karasev A. V. Boyko V. P. Gowda S. Nikolaeva O. V. Hilf M. E. Koonin E. V.. (1995). Complete sequence of the *Citrus tristeza virus* RNA genome. Virology 208, 511–520. 10.1006/viro.1995.1182 7747424

[B43] Kumar N. Bera B. C. Greenbaum B. D. Bhatia S. Sood R. Selvaraj P.. (2016). Revelation of influencing factors in overall codon usage bias of equine influenza viruses. PLoS ONE 11:e0154376. 10.1371/journal.pone.0154376 27119730 PMC4847779

[B44] Kyriakou A. Ioannou N. Gavriel J. Bar-Joseph M. Papayiannis C. Kapar-Isaia T.. (1996). “Management of *Citrus tristeza virus* in Cyprus,” in Proceedings of the Fifteenth Conference of the International Organization of Citrus Virologists, eds. J. V. Da Graça, P. Moreno, R. K. Yokomi (IOCV, CA: Riverside), 172–178. 10.5070/C560F22132

[B45] Lattanzio V. Lattanzio V. M. T. Cardinali A. (2006). “Role of phenolics in the resistance mechanisms of plants against fungal pathogens and insects,” in Phytochemistry: Advances in Research, ed. F. Imperato, (Research Signpost, Kerala, India), 23–67.

[B46] Lee R. F. Brlansky R. H. Garnsey S. M. Yokomi R. K. (1987). Traits of *Citrus tristeza virus* important for mild strain cross protection of citrus: the Florida approach. Phytophylactica 19, 215–218.

[B47] Lee R. F. Keremane M. L. (2013). Mild strain cross protection of tristeza: a review of research to protect against decline on sour orange in Florida. Front. Microbiol. 4:259. 10.3389/fmicb.2013.00259 24046764 PMC3764332

[B48] Ligges U. M. M. (2003). Scatterplot3d – an R package for visualizing multivariate data. J. Stat. Softw. 8, 1–20. 10.18637/jss.v008.i11

[B49] Link K. Walker J. C. (1933). The isolation of catechol from pigmented onion scales and its significance in relation to disease resistance in onion. J. Biol. Chem. 100, 379–383. 10.1016/S0021-9258(18)75955-3

[B50] Liu Y. Q. Heying E. Tanumihardjo S. A. (2012). History, global distribution and nutritional importance of citrus fruits. Compr. Rev. Food Sci. Food Saf. 11, 530–545. 10.1111/j.1541-4337.2012.00201.x

[B51] Livak K. J. Schmittgen T. D. (2001). Analysis of relative gene expression data using real-time quantitative PCR and the 2(-Delta Delta C(T)) method. Methods 25, 402–408. 10.1006/meth.2001.1262 11846609

[B52] Malik E. P. Singh M. B. (1980). Plant Enzymology and Histoenzymology. 1st Edn. Kalyani Publishers, New Delhi, 286.

[B53] Masaló I. and Oca J. (2020). Evaluation of a portable chlorophyll optical meter to estimate chlorophyll concentration in the green seaweed *Ulva ohnoi*. J. Appl. Phycol. 32, 4171–4174. 10.1007/s10811-020-02257-3

[B54] Melzer M. J. Borth W. B. Sether D. M. Ferreira S. Gonsalves D. Hu J. S. (2010). Genetic diversity and evidence for recent modular recombination in Hawaiian *Citrus tristeza virus*. Virus Genes 40, 111–118. 10.1007/s11262-009-0409-3 19834797

[B55] Moreno P. Guerri J. Ballester-Olmos J. F. Albiach R. Martínez M. E. (1993). Separation and interference of strains from a *Citrus tristeza virus* isolate evidenced by biological activity and double-stranded RNA (dsRNA) analysis. Plant Pathol. 42, 35–41. 10.1111/j.1365-3059.1993.tb01469.x

[B56] Mueller S. Papamichail D. Coleman J. R. Skiena S. Wimmer E. (2006). Reduction of the rate of poliovirus protein synthesis through large-scale codon deoptimization causes attenuation of viral virulence by lowering specific infectivity. J. Virol. 80, 9687–9696. 10.1128/JVI.00738-06 16973573 PMC1617239

[B57] Muhire B. M. Varsani A. Martin D. P. (2014). SDT: a virus classification tool based on pairwise sequence alignment and identity calculation. PLoS ONE 9:e108277. 10.1371/journal.pone.0108277 25259891 PMC4178126

[B58] Müller G. W. Rezende J. A. (2004). Preimmunization: applications and perspectives in virus disease control. In *Diseases of Fruits and Vegetables, Vol. 1*.ed. S. A. M. H. Naqvi (Netherlands: Springer), 361–395. 10.1007/1-4020-2606-4_9

[B59] Nakamura Y. Gojobori T. Ikemura T. (2000). Codon usage tabulated from international DNA sequence databases: status for the year 2000. Nucleic Acids Res. 28:292. 10.1093/nar/28.1.292 10592250 PMC102460

[B60] Navarro L. Pina J. A. Juárez J. Ballester-Olmos J. F. Arregui J. M. Ortega C.. (2002). “The citrus variety improvement program in Spain in the period 1975–2001,” in Proceedings of the Fifteenth Conference of the International Organization of Citrus Virologists,eds. N. Duran-Vila, R. G. Milne,J. V. Da Graça (IOCV, CA: Riverside), 306–316. 10.5070/C542M9S93V

[B61] Plotkin J. B. Kudla G. (2011). Synonymous but not the same: the causes and consequences of codon bias. Nat. Rev. Genet. 12, 32–42. 10.1038/nrg2899 21102527 PMC3074964

[B62] Plummer D. T. (1990). An Introduction to Practical Biochemistry, 3rd Edn. New Delhi: Tata McGraw-Hill Publishing Company.

[B63] Rahman S. U. Yao X. Li X. Chen D. Tao S. (2017). Analysis of codon usage bias of Crimean-Congo hemorrhagic fever virus and its adaptation to hosts. Infect. Genet. Evol. 58, 1–16. 10.1016/j.meegid.2017.11.027 29198972

[B64] Rashad Y. Aseel D. Hammad S. (2020). “Phenolic compounds against fungal and viral plant diseases,” in Plant Phenolics in Sustainable Agriculture (Singapore: Springer). 10.1007/978-981-15-4890-1_9

[B65] Riedle-Bauer M. (2000). Role of reactive oxygen species and antioxidant enzymes in systemic virus infections of plants. J. Phytopathol. 148, 297–302. 10.1046/j.1439-0434.2000.00503.x

[B66] Rocha-Peña M. A. Lee R. F. Lastra R. Niblett C. L. Ochoa-Corona F. M. Garnsey S. M.. (1995). *Citrus tristeza virus* and its aphid vector *Toxoptera citricida*: threats to citrus production in the Caribbean and Central and North America. Plant Dis. 79, 437–445. 10.1094/PD-79-0437

[B67] Roy A. Brlansky R. H. (2010). Genome analysis of an orange stem pitting *Citrus tristeza virus* isolate reveals a novel recombinant genotype. Virus Res. 151, 118–130. 10.1016/j.virusres.2010.03.017 20381550

[B68] Sharp P. M. Li W. H. (1986). An evolutionary perspective on synonymous codon usage in unicellular organisms. J. Mol. Biol. 24, 28–38. 10.1007/BF02099948 3104616

[B69] Sheen J. Zhou L. Jang J. C. (1999). Sugars as signaling molecules. Curr. Opin. Plant Biol. 2, 410–418. 10.1016/S1369-5266(99)00014-X 10508760

[B70] Simón D. Fajardo A. Sonora M. Delfraro A. Musto H. (2017). Host influence in the genomic composition of flaviviruses: a multivariate approach. Biochem. Biophys. Res. Commun. 492, 572–578. 10.1016/j.bbrc.2017.06.088 28630001

[B71] Singh Y. J. Grewal S. K. Gill R. K. (2022). Role of antioxidative defense in yellow mosaic disease resistance in black gram (*Vigna mungo* (L.) Hepper). J. Plant Growth Regul. 41, 2138–2156. 10.1007/s00344-021-10431-1

[B72] Smeekens S. J. A. (2000). Sugar-induced signal transduction in plants. Annu. Rev. Plant Biol. 51, 49–81. 10.1146/annurev.arplant.51.1.49 15012186

[B73] Stoletzki N. Eyre-Walker A. (2007). Synonymous codon usage in *Escherichia coli*: selection for translational accuracy. Mol. Biol. Evol. 24, 374–381. 10.1093/molbev/msl166 17101719

[B74] Tamura K. Stecher G. Kumar S. (2021). MEGA11: molecular evolutionary genetics analysis version 11. Mol. Biol. Evol. 38, 3022–3027. 10.1093/molbev/msab120 33892491 PMC8233496

[B75] Tarafdar A. Godara S. Dwivedi S. Jayakumar B. K. Biswas K. K. (2013). Characterization of *Citrus tristeza virus* and determination of genetic variability in North-east and South India. Indian Phytopathol. 66, 302–307.

[B76] Thompson J. D. Gibson T. J. Plewniak F. Jeanmougin F. Higgins D. G. (1997). The clustal X windows interface: flexible strategies for multiple sequence alignment aided by quality analysis tools. Nucleic Acids Res. 25, 4876–4882. 10.1093/nar/25.24.4876 9396791 PMC147148

[B77] Tian L. Shen X. Murphy R. W. Shen Y. (2018). The adaptation of codon usage of +ssRNA viruses to their hosts. Infect. Genet. Evol. 63, 175–179. 10.1016/j.meegid.2018.05.034 29864509 PMC7106036

[B78] Tsai C. T. Lin C. H. Chang C. Y. (2007). Analysis of codon usage bias and base compositional constraints in iridovirus genomes. Virus Res. 126, 196–206. 10.1016/j.virusres.2007.03.001 17434639

[B79] Van Bueren E. T. L. Struik P. C. van Eekeren N. Nuijten E. (2018). Towards resilience through systems-based plant breeding: a review. Agron. Sustain. Dev. 38:42. 10.1007/s13593-018-0522-6 30956692 PMC6417397

[B80] Vance V. Vaucheret H. (2001). RNA silencing in plants: defense and counterdefense. Science 292, 2277–2280. 10.1126/science.1061334 11423650

[B81] Wang Q. Lyu X. Cheng J. Fu Y. Lin Y. Abdoulaye A. H.. (2022). Codon usage provides insights into the adaptive evolution of mycoviruses in their associated fungi host. Int. J. Mol. Sci. 23:7441. 10.3390/ijms23137441 35806445 PMC9267111

[B82] Wang X. Jiang Z. Yue N. Jin X. Zhang X. Li Z.. (2021). *Barley stripe mosaic virus* γb protein disrupts chloroplast antioxidant defenses to optimize viral replication. EMBO J. 40:e107660. 10.15252/embj.2021107660 34254679 PMC8365260

[B83] Wang X. Xu W. Fan K. Chiu H. C. Huang C. (2020). Codon usage bias in the H gene of canine distemper virus. Microb. Pathog. 149, 104511. 10.1016/j.micpath.2020.104511 32961282

[B84] Xu X. Z. Liu Q. P. Fan L. J. Cui X. F. Zhou X. P. (2008). Analysis of synonymous codon usage and evolution of begomoviruses. J. Zhejiang Univ. Sci. B 9, 667–674. 10.1631/jzus.B0820005 18763298 PMC2528880

[B85] Yokomi R. Selvaraj V. Maheshwari Y. Chiumenti M. Saponari M. Giampetruzzi A.. (2018). Molecular and biological characterization of a novel mild strain of *Citrus tristeza virus* in California. Arch. Virol. 163, 1795–1804. 10.1007/s00705-018-3799-5 29550931

