# Supplementary material for: Differentiation and validation of mild and severe strains of citrus tristeza virus through codon usage bias, host adaptation, and biochemical profiling
Source: Front Microbiol. 2025 Oct 1;16:1665893. doi: 10.3389/fmicb.2025.1665893 (PMC12521140; doi:10.3389/fmicb.2025.1665893)
Supplement: Supplementary file 1 [file Data_Sheet_1.zip › Datasheet 1/Presentation 1 (56).PPTX]

## Slide 1
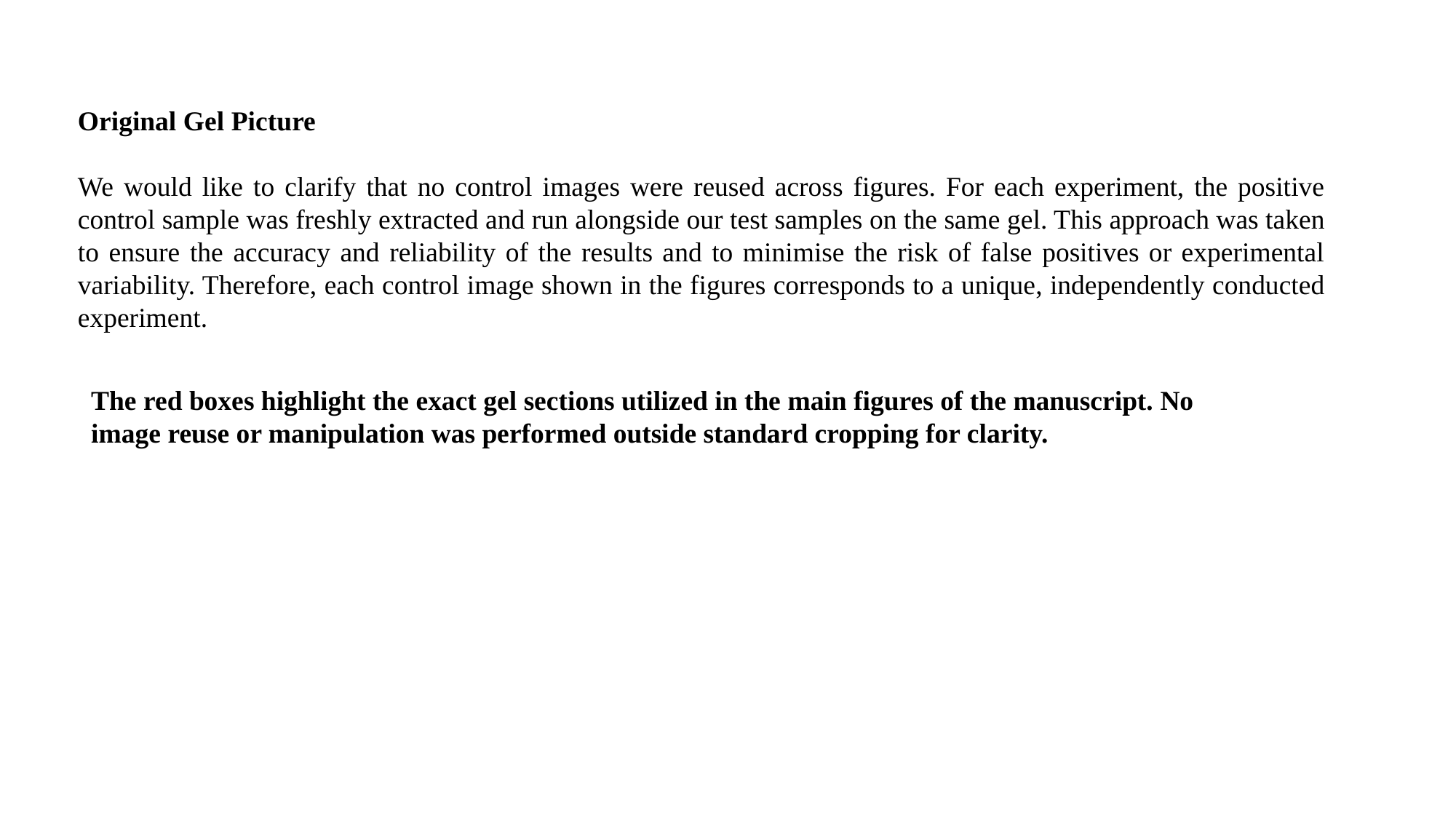

Original Gel Picture
We would like to clarify that no control images were reused across figures. For each experiment, the positive control sample was freshly extracted and run alongside our test samples on the same gel. This approach was taken to ensure the accuracy and reliability of the results and to minimise the risk of false positives or experimental variability. Therefore, each control image shown in the figures corresponds to a unique, independently conducted experiment.
The red boxes highlight the exact gel sections utilized in the main figures of the manuscript. No image reuse or manipulation was performed outside standard cropping for clarity.

## Slide 2
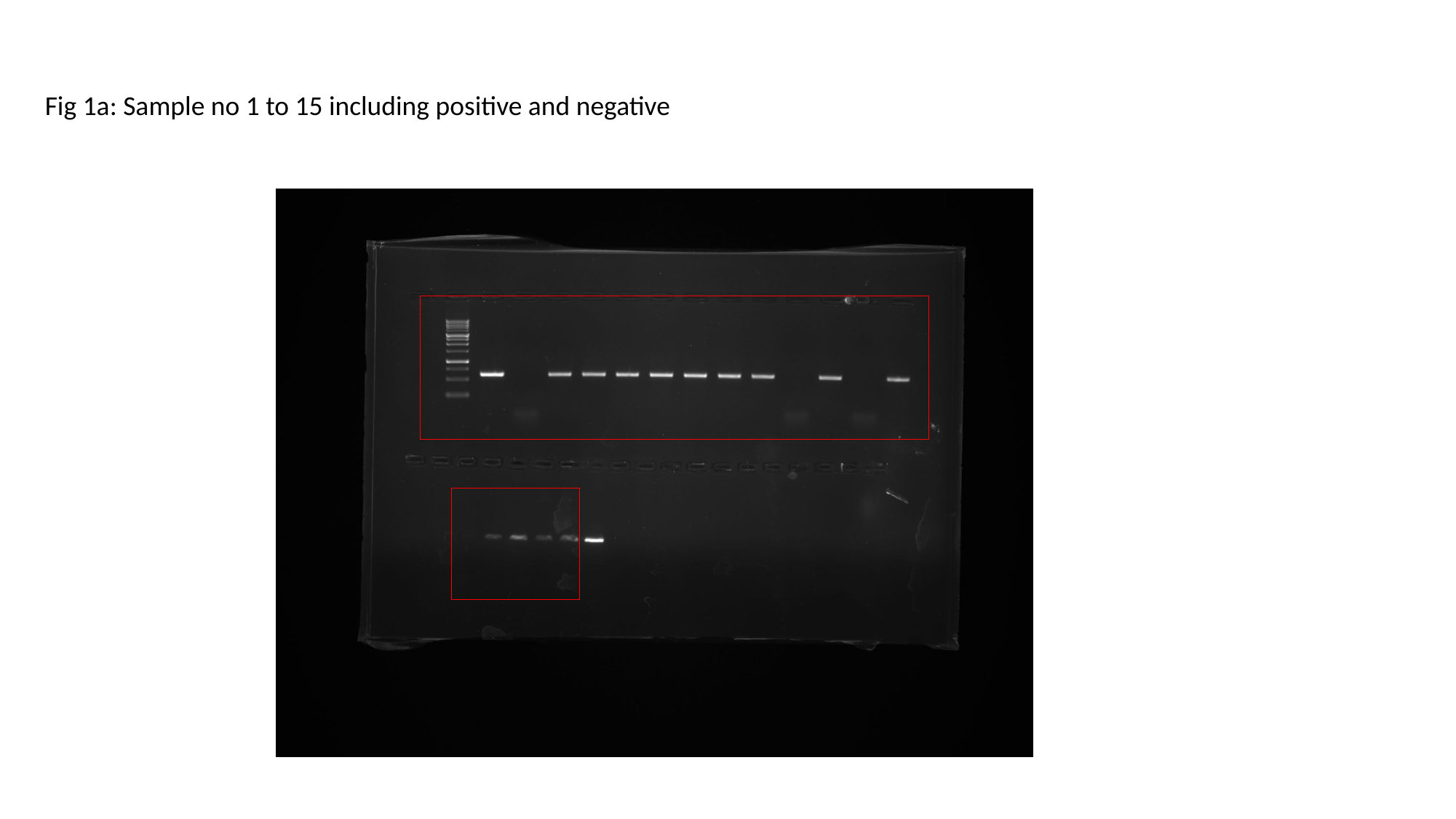

Fig 1a: Sample no 1 to 15 including positive and negative

## Slide 3
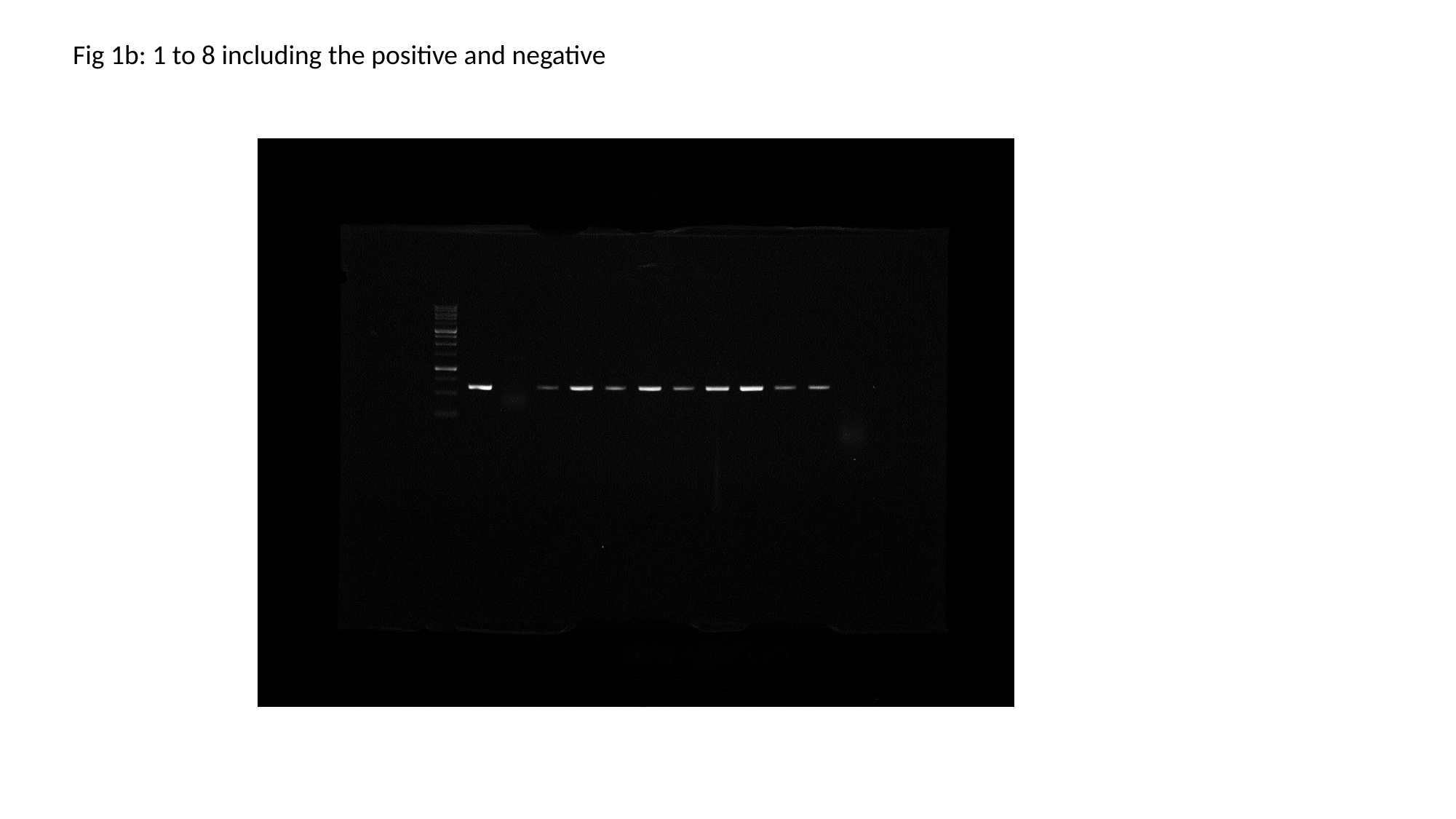

Fig 1b: 1 to 8 including the positive and negative

## Slide 4
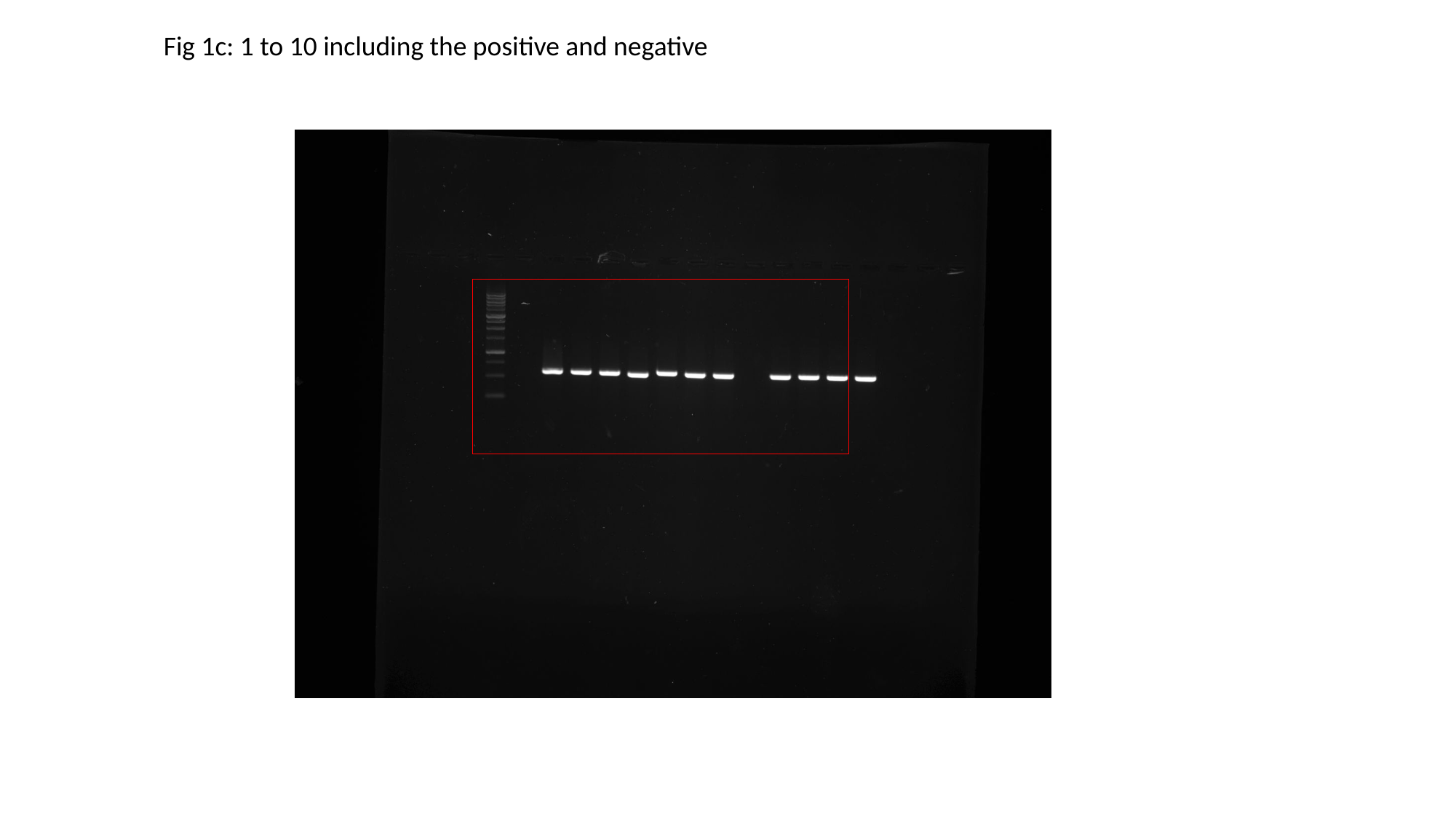

Fig 1c: 1 to 10 including the positive and negative

## Slide 5
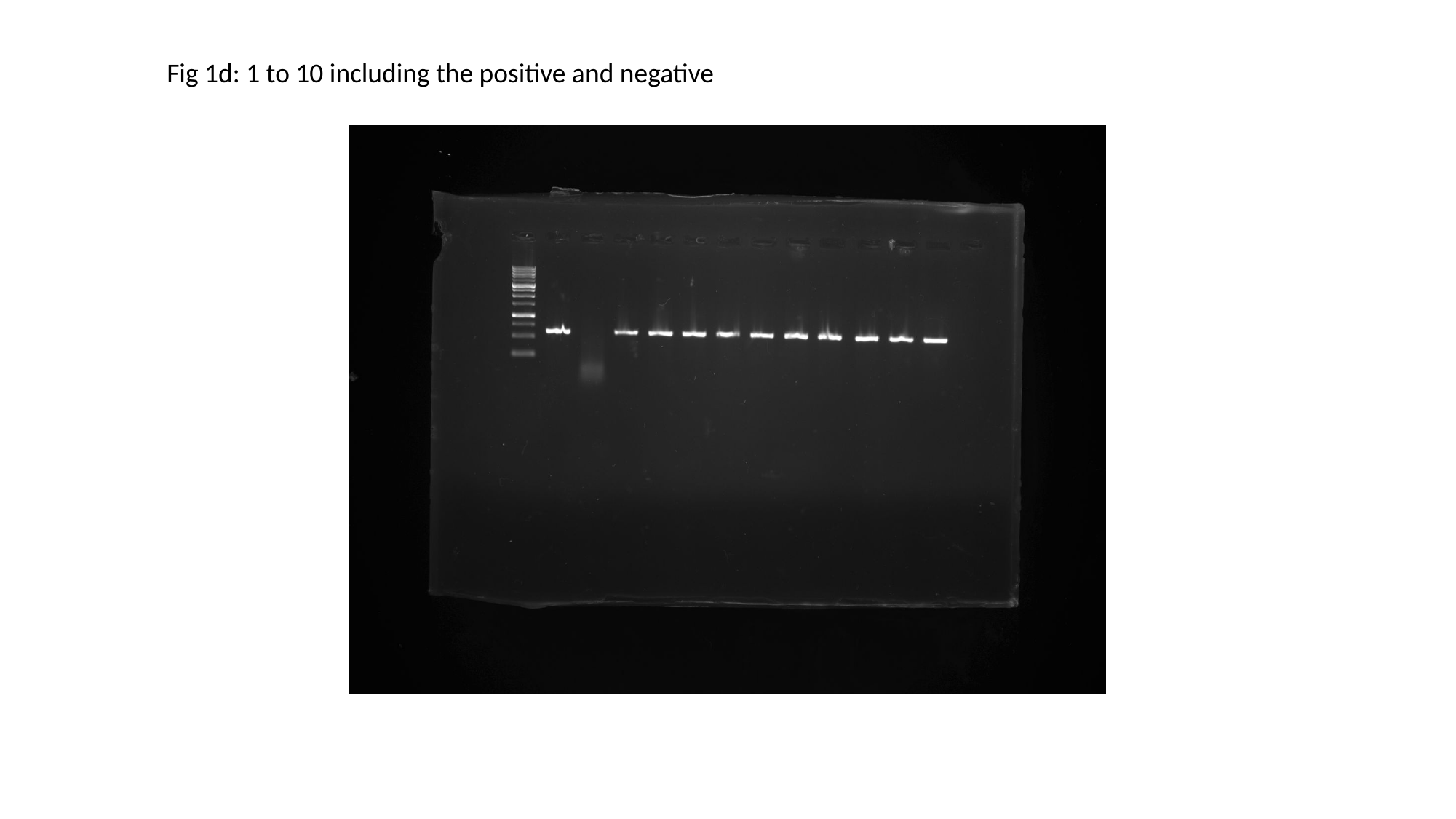

Fig 1d: 1 to 10 including the positive and negative

## Slide 6
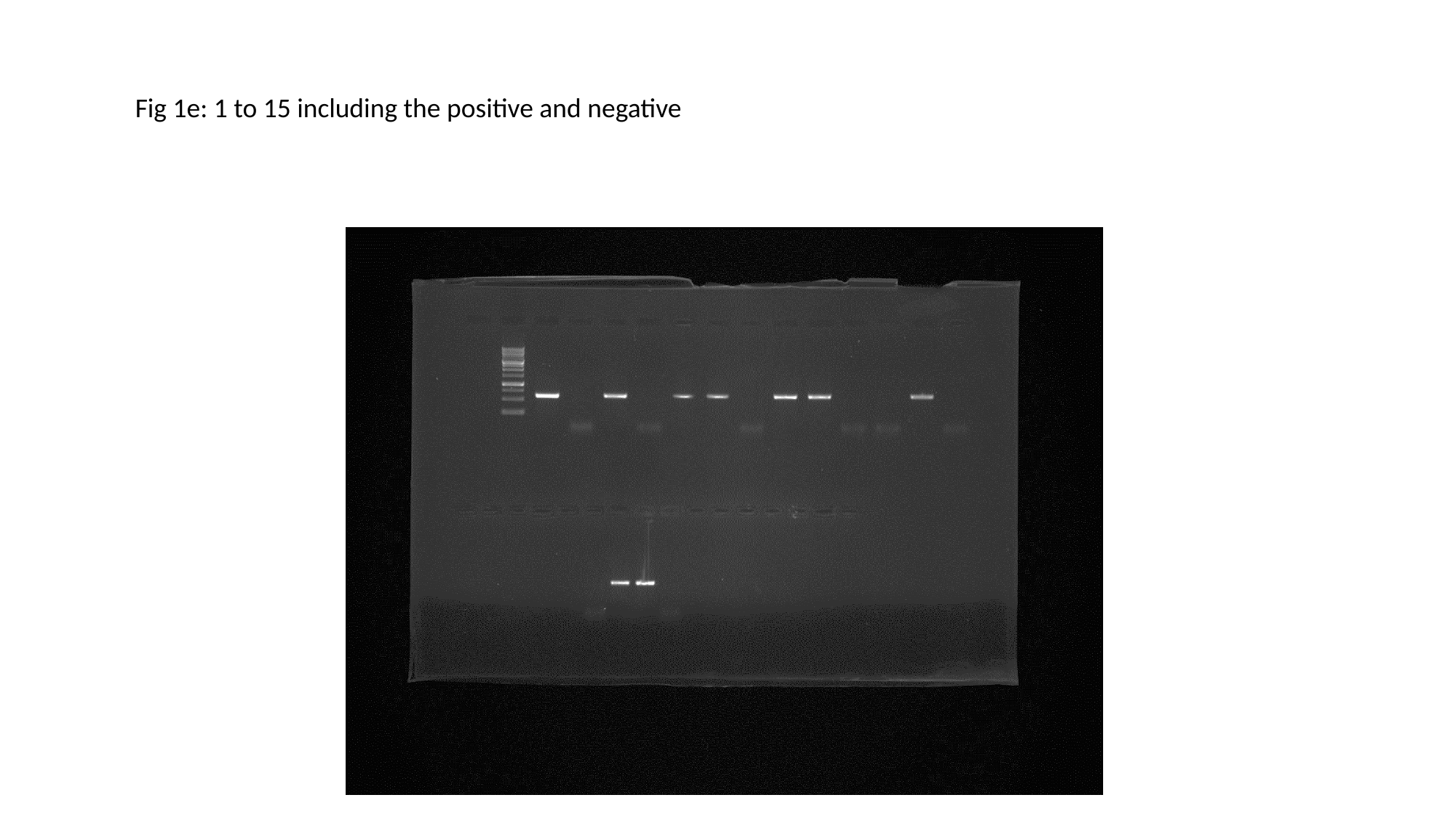

Fig 1e: 1 to 15 including the positive and negative
