# Supplementary material for: Differentiation and validation of mild and severe strains of citrus tristeza virus through codon usage bias, host adaptation, and biochemical profiling
Source: Front Microbiol. 2025 Oct 1;16:1665893. doi: 10.3389/fmicb.2025.1665893 (PMC12521140; doi:10.3389/fmicb.2025.1665893)
Supplement: Supplementary file 1 [file Data_Sheet_1.zip › Datasheet 1/Supplementary Tables 1-6.docx]

**TABLE S1 Primer sequences, expected amplicon size and genetic region amplified in PCR assays.**

| **Primer name** | **Primer sequence (5’–3’)** | **Amplicon size (bp)** | **Annealing temp. (°C)** | **Genetic Region amplified** | **Reference** |
| --- | --- | --- | --- | --- | --- |
| CP11F | ATGGACGACGARACWAAGAA | 672 | 59 | Coat protein gene | Design in this study |
| CP11R | TCAACGTGTGTTAAATTTCCCA |  |  |  |  |

**TABLE S2** **List of CTV Isolates Used for Codon Usage Bias Analysis, Including the Present Study, Indian Isolates, and International Isolates.**

| **S.no** | **Isolates** | **Region** | **Source Host** | **CP Accession No.** |
| --- | --- | --- | --- | --- |
| 1 | BHKM-1 | Assam (Boko) | *Citrus reticulata* | PQ219874 |
| 2 | BHKM-2 | Assam (Boko) | *Citrus reticulata* | PQ219875 |
| 3 | BHKM-3 | Assam (Boko) | *Citrus reticulata* | PQ219876 |
| 4 | BHKM-4 | Assam (Boko) | *Citrus reticulata* | PQ219877 |
| 5 | BHKM-6 | Assam (Boko) | *Citrus reticulata* | PQ219878 |
| 6 | BHKM-7 | Assam (Boko) | *Citrus reticulata* | PQ219879 |
| 7 | BHKM-9 | Assam (Boko) | *Citrus reticulata* | PQ219880 |
| 8 | DeKM-1 | Assam(Dhupdhara Goalpara) | *Citrus reticulata* | PQ219881 |
| 9 | DeKM-2 | Assam(Dhupdhara Goalpara) | *Citrus reticulata* | PQ219882 |
| 10 | DeKM-3 | Assam(Dhupdhara Goalpara) | *Citrus reticulata* | PQ219883 |
| 11 | DeKM-4 | Assam(Dhupdhara Goalpara) | *Citrus reticulata* | PQ219884 |
| 12 | DeKM-5 | Assam(Dhupdhara Goalpara) | *Citrus reticulata* | PQ219885 |
| 13 | DeKM-6 | Assam(Dhupdhara Goalpara) | *Citrus reticulata* | PQ219886 |
| 14 | DeKM-7 | Assam(Dhupdhara Goalpara) | *Citrus reticulata* | PQ219887 |
| 15 | DeKM-8 | Assam(,Dhupdhara Goalpara) | *Citrus reticulata* | PQ219888 |
| 16 | DeKM-9 | Assam(Dhupdhara Goalpara) | *Citrus reticulata* | PQ219889 |
| 17 | DeKM-10 | Assam(Dhupdhara Goalpara) | *Citrus reticulata* | PQ219890 |
| 18 | GDKM-1 | Assam(Dhupdhara Goalpara) | *Citrus reticulata* | PQ219891 |
| 19 | GDKM-2 | Assam(Dhupdhara Goalpara) | *Citrus reticulata* | PQ219892 |
| 20 | GDKM-3 | Assam(Dhupdhara Goalpara) | *Citrus reticulata* | PQ219893 |
| 21 | GDKM-4 | Assam(Dhupdhara Goalpara) | *Citrus reticulata* | PQ219894 |
| 22 | GDKM-5 | Assam(Dhupdhara Goalpara) | *Citrus reticulata* | PQ219895 |
| 23 | GDKM-6 | Assam(,Dhupdhara Goalpara) | *Citrus reticulata* | PQ219896 |
| 24 | GDKM-8 | Assam(Dhupdhara Goalpara) | *Citrus reticulata* | PQ219897 |
| 25 | GDKM-9 | Assam(Dhupdhara Goalpara) | *Citrus reticulata* | PQ219898 |
| 26 | GDKM-10 | Assam(Dhupdhara Goalpara) | *Citrus reticulata* | PQ219899 |
| 27 | KKM-2 | Assam(Dhupdhara Goalpara) | *Citrus reticulata* | PQ219900 |
| 28 | KKM-4 | Assam(Dhupdhara Goalpara) | *Citrus reticulata* | PQ219901 |
| 29 | KKM-5 | Assam(Dhupdhara Goalpara) | *Citrus reticulata* | PQ219902 |
| 30 | KKM-7 | Assam(Dhupdhara Goalpara) | *Citrus reticulata* | PQ219903 |
| 31 | KKM-8 | Assam(Dhupdhara Goalpara) | *Citrus reticulata* | PQ219904 |
| 32 | KKM-14 | Assam(Dhupdhara Goalpara) | *Citrus reticulata* | PQ219905 |
| 33 | KKM-32 | Assam(Dhupdhara Goalpara) | *Citrus reticulata* | PQ219906 |
| 34 | KKM-33 | Assam(Dhupdhara Goalpara) | *Citrus reticulata* | PQ219907 |
| 35 | RTKM-1 | Assam(Kolangpur,Kamrup Metro) | *Citrus reticulata* | PQ219908 |
| 36 | RTKM-2 | Assam(Kolangpur,Kamrup Metro) | *Citrus reticulata* | PQ219909 |
| 37 | ASKM-1 | Assam( Kolangpur,Kamrup Metro) | *Citrus reticulata* | PQ219910 |
| 38 | ASKM-2 | Assam( Kolangpur,Kamrup Metro) | *Citrus reticulata* | PQ219911 |
| 39 | MKM-2 | Assam(Kolangpur,Kamrup Metro) | *Citrus reticulata* | PQ219912 |
| 40 | KTKM-3 | Assam(Kolangpur,Kamrup Metro) | *Citrus reticulata* | PQ219913 |
| 41 | KTKM-4 | Assam(Kolangpur,Kamrup Metro) | *Citrus reticulata* | PQ219914 |
| 42 | SRKM-1 | Assam(Kolangpur,Kamrup Metro) | *Citrus reticulata* | PQ219915 |
| 43 | SRKM-2 | Assam(Kolangpur,Kamrup Metro) | *Citrus reticulata* | PQ219916 |
| 44 | SRKM-4 | Assam(Kolangpur,Kamrup Metro) | *Citrus reticulata* | PQ219917 |
| 45 | SRKM-5 | Assam(Kolangpur,Kamrup Metro) | *Citrus reticulata* | PQ219918 |
| 46 | SRKM-6 | Assam(Kolangpur,Kamrup Metro) | *Citrus reticulata* | PQ219919 |
| 47 | AKM-1 | Assam(Kolangpur,Kamrup Metro) | *Citrus reticulata* | PQ219920 |
| 48 | CTV-P2 | Vidarbha(Maharashtra) | *Citrus aurantifolia* | GQ475564 |
| 49 | CTV-P3 | Vidarbha(Maharashtra) | *Citrus aurantifolia* | GQ475565 |
| 50 | CTV-P4 | Vidarbha(Maharashtra) | *Citrus aurantifolia* | GQ475566 |
| 51 | CTV-P7 | Vidarbha(Maharashtra) | *Citrus sinensis* | GQ475567 |
| 52 | CTV-KAT1 | Vidarbha(Maharashtra) | *Citrus sinensis* | GQ47557 |
| 53 | CTV-KAT2 | Vidarbha(Maharashtra) | *Citrus sinensis* | GQ47558 |
| 54 | CTV-KAT3 | Vidarbha(Maharashtra) | *Citrus sinensis* | GQ47559 |
| 55 | CTV-KAT4 | Vidarbha(Maharashtra) | *Citrus sinensis* | GQ47560 |
| 56 | CTV-KAT5 | Vidarbha(Maharashtra) | *Citrus reticulata* | GQ47561 |
| 57 | CTV-KAT6 | Vidarbha(Maharashtra) | *Citrus reticulata* | GQ47562 |
| 58 | CTV-KAT7 | Vidarbha(Maharashtra) | *Citrus sinensis* | KC694143 |
| 59 | CTV-Kpg1 | Kalimpong(West bengal) | *Citrus aurantifolia* | GQ392062 |
| 60 | CTV-Kpg2 | Kalimpong(West bengal) | *Citrus aurantifolia* | GQ228821 |
| 61 | CTV-Kpg3 | Kalimpong(West bengal) | *Citrus reticulata* | HM573451 |
| 62 | CTV-K2 | Kalimpong(West bengal) | *Citrus reticulata* | GQ475540 |
| 63 | CTV-K4 | Kalimpong(West bengal) | *Citrus aurantifolia* | GQ475541 |
| 64 | CTV-K5 | Kalimpong(West bengal) | *Citrus aurantifolia* |  |
| 65 | CTV-K6 | Kalimpong(West bengal) | *Citrus aurantifolia* | GQ475542 |
| 66 | CTV-K7 | Kalimpong(West bengal) | *Citrus aurantifolia* | GQ475543 |
| 67 | CTV-K8 | Kalimpong(West bengal) | *Citrus aurantifolia* | GQ475544 |
| 68 | CTV-K9 | Kalimpong(West bengal) | *Citrus aurantifolia* |  |
| 69 | CTV-K10 | Kalimpong(West bengal) | *Citrus aurantifolia* |  |
| 70 | CTV-K11 | Kalimpong(West bengal) | *Citrus aurantifolia* | GQ475545 |
| 71 | CTV-K13 | Kalimpong(West bengal) | *Citrus aurantifolia* |  |
| 72 | CTV-K16 | Kalimpong(West bengal) | *Citrus aurantifolia* | GQ475547 |
| 73 | CTV-K17 | Kalimpong(West bengal) | *Citrus aurantifolia* | GQ475548 |
| 74 | CTV-K18 | Kalimpong(West bengal) | *Citrus aurantifolia* |  |
| 75 | CTV-K20 | Kalimpong(West bengal) | *Citrus aurantifolia* |  |
| 76 | CTV-D3 | Delhi | *Citrus aurantifolia* | FJ001821 |
| 77 | CTV-D4 | Delhi | *Citrus aurantifolia* | FJ001822 |
| 78 | CTV-D5 | Delhi | *Citrus sinensis* | GQ475538 |
| 79 | CTV-D6 | Delhi | *Citrus sinensis* | FJ001823 |
| 80 | CTV-D7 | Delhi | *Citrus sinensis* | FJ001824 |
| 81 | CTV-D8 | Delhi | *Citrus sinensis* | GQ475539 |
| 82 | CTV-D10 | Delhi | *Citrus sinensis* | FJ001826 |
| 83 | CTV-D11 | Delhi | *Citrus sinensis* | FJ001827 |
| 84 | CTV-D12 | Delhi | *Citrus sinensis* | FJ001828 |
| 85 | CTV-D13 | Delhi | *Citrus sinensis* | FJ001829 |
| 86 | CTV-D14 | Delhi | *Citrus sinensis* | FJ001830 |
| 87 | CTV-D15 | Delhi | *Citrus sinensis* | FJ001831 |
| 88 | CTV-TP1 | Tirupati(Andhra Pardesh) | *Citrus sinensis* | EF460127 |
| 89 | CTV-TP3 | Tirupati(Andhra Pardesh) | *Citrus sinensis* | KC590501 |
| 90 | CTV-TP4 | Tirupati(Andhra Pardesh) | *Citrus aurantifolia* | KC590502 |
| 91 | CTV-TP5 | Tirupati(Andhra Pardesh) | *Citrus aurantifolia* | KC590503 |
| 92 | CTV-TP6 | Tirupati(Andhra Pardesh) | *Citrus sinensis* | KC590504 |
| 93 | CTV-TP7 | Tirupati(Andhra Pardesh) | *Citrus sinensis* | KC590505 |
| 94 | CTV-TP9 | Tirupati(Andhra Pardesh) | *Citrus limettioides* | KC590500 |
| 95 | CTV-TP11 | Tirupati(Andhra Pardesh) | *Citrus sinensis* | KC590508 |
| 96 | CTV-TP12 | Tirupati(Andhra Pardesh) | *Citrus sinensis* | KC590509 |
| 97 | AR2 | Assam | *Citrus aurantifolia* | KC590489 |
| 98 | AR13 | Assam | *Citrus reticulata* | KC590490 |
| 99 | AR16 | Assam | *Citrus sinensis* | KC590491 |
| 100 | MB3 | Assam | *Citrus sinensis* | KC590492 |
| 101 | MB7 | Assam | *Citrus reticulata* | KC590496 |
| 102 | AG26 | Assam | *Citrus reticulata* | KC590497 |
| 103 | AG28 | Assam | *Citrus reticulata* | KC590498 |
| 104 | N1 | Assam | *Citrus aurantifolia* | KC986384 |
| 105 | Mnp1 | Assam | *Citrus reticulata* | KU530134 |
| 106 | Mnp2 | Assam | *Citrus reticulata* | KU530135 |
| 107 | Mnp3 | Assam | *Citrus reticulata* | KU530136 |
| 108 | VT (VT type) | Israel | *C. sinensis* | U56902 |
| 109 | T36 (T36 type) | FL (USA) | *C. sinensis* | UL6304 |
| 110 | T30 (T30 type) | FL (USA) | *C. sinensis* | AF260651 |
| 111 | T3 (T3 type) | FL (USA) | *C. sinensis*  (Sweet orange | KC525952 |
| 112 | NZRB-T30 (RB Type) | New Zealand | *Poncirus trifoliata* (Trifoliate orange) | FJ525434 |
| 113 | B165 |  |  |  |

**TABLE S3** **Source of Khasi mandarin twigs collected from Assam and maintained in the Glasshouse, IARI, New Delhi.**

| Sn. | Lane no | Sample | District | Village | Age | Symptoms | PCR amplification |
| --- | --- | --- | --- | --- | --- | --- | --- |
| 1 | 1 | SRKM-5 | Kamrup Metro | Kolangpur | 66-67 | H,Mg | + |
| 2 | 2 | RTKM-1 | Kamrup Metro | Kolangpur | 72-74 | H,Mg | + |
| 3 | 3 | MKM-2 | Kamrup Metro | Kolangpur | 72-74 | H,LY | + |
| 4 | 4 | AKM-1 | Kamrup Metro | Kolangpur | 67-68 | H,Mg | + |
| 5 | 5 | SRKM-1 | Kamrup Metro | Kolangpur | 68-69 | H,Mg | + |
| 6 | 6 | SRKM-2 | Kamrup Metro | Kolangpur | 62-63 | H, Gg | + |
| 7 | 7 | SRKM-4 | Kamrup Metro | Kolangpur | 62-63 | H,Mg | + |
| 8 | 8 | *MKM-1 | Kamrup Metro | Kolangpur | 73-74 | H,Gg | - |
| 9 | 9 | SRKM-6 | Kamrup Metro | Kolangpur | 73-74 | H,L,Mg | + |
| 10 | 10 | *MKM-3 | Kamrup Metro | Kolangpur | 65-66 | H,Gg | - |
| 11 | 11 | RTKM-2 | Kamrup Metro | Kolangpur | 73-74 | H, Mg | + |
| 12 | 12 | KTKM-3 | Karbi Anglong | Kolangpur | 72-74 | H,Mg | + |
| 13 | 13 | KTKM-4 | Karbi Anglong | Kolangpur | 65-66 | H,Mg | + |
| 14 | 14 | ASKM-1 | Kamrup Metro | Kolangpur | 67-68 | H,Mg | + |
| 15 | 15 | ASKM-2 | Kamrup Metro | Kolangpur | 68-69 | H,Mg | + |
| 16 | 1 | BHKM-1 | Kamrup Rural | Boko | 69-70 | H,Mg | + |
| 17 | 2 | BHKM-2 | Kamrup Rural | Boko | 68-69 | H,Mg | + |
| 18 | 3 | BHKM-3 | Kamrup Rural | Boko | 69-70 | H,Mg | + |
| 19 | 4 | BHKM-4 | Kamrup Rural | Boko | 64-65 | H,Mg | + |
| 20 | 5 | BHKM-6 | Kamrup Rural | Boko | 66-67 | H,Mg | + |
| 21 | 6 | BHKM-7 | Kamrup Rural | Boko | 60-61 | H,Mg | + |
| 22 | 7 | BHKM-9 | Kamrup Rural | Boko | 62-63 | H,Mg | + |
| 23 | 8 | BHKM-10 | Kamrup Rural | Boko | 69-70 | H,GG | - |
| 24 | 1 | GDKM-1 | Goalpara | SNB, Dhupdhara | 66-68 | H,Mg | + |
| 25 | 2 | GDKM-2 | Goalpara | SNB,Dhupdhara | 67-68 | H,Mg | + |
| 26 | 3 | GDKM-3 | Goalpara | SNB,Dhupdhara | 60-61 | H,Mg | + |
| 27 | 4 | GDKM-4 | Goalpara | SNB,Dhupdhara | 61-62 | H,Mg | + |
| 28 | 5 | GDKM-5 | Goalpara | SNB,Dhupdhara | 70-72 | H,Mg | + |
| 29 | 6 | GDKM-6 | Goalpara | SNB,Dhupdhara | 76-78 | H,Mg | + |
| 30 | 7 | *GDKM-7 | Goalpara | SNB, Dhupdhara | 75-76 | H,Gg | - |
| 31 | 8 | GDKM-8 | Goalpara | SNB, Dhupdhara | 73-74 | H,Mg | + |
| 32 | 9 | GDKM-9 | Goalpara | SNB, Dhupdhara | 72-73 | H,Mg | + |
| 33 | 10 | GDKM-10 | Goalpara | SNB, Dhupdhara | 77-78 | H,Mg | + |
| 34 | 1 | DeKM-1 | Goalpara | SNB, Dhupdhara | 73-74 | H,LY | + |
| 35 | 2 | DeKM-2 | Goalpara | SNB, Dhupdhara | 72-74 | H,Mg | + |
| 36 | 3 | DeKM-3 | Goalpara | SNB, Dhupdhara | 65-66 | H,Mg | + |
| 37 | 4 | DeKM-4 | Goalpara | SNB, Dhupdhara | 67-68 | H, Mg | + |
| 38 | 5 | DeKM-5 | Goalpara | SNB, Dhupdhara | 68-69 | H,Mg | + |
| 39 | 6 | DeKM-6 | Goalpara | SNB, Dhupdhara | 62-63 | H,LY | + |
| 40 | 7 | DeKM-7 | Goalpara | SNB, Dhupdhara | 61-63 | H,Mg | + |
| 41 | 8 | DeKM-8 | Goalpara | SNB, Dhupdhara | 62-63 | H,Mg | + |
| 42 | 9 | DeKM-9 | Goalpara | SNB, Dhupdhara | 66-67 | H,Mg | + |
| 43 | 10 | DeKM-1 | Goalpara | SNB, Dhupdhara | 73-74 | H,Mg | + |
| 44 | 1 | KKM-2 | Goalpara | SNB, Dhupdhara | 65-66 | H,VMg | + |
| 45 | 2 | *KKM-3 | Goalpara | SNB, Dhupdhara | 67-68 | H,Gg | - |
| 46 | 3 | KKM-4 | Goalpara | SNB, Dhupdhara | 68-69 | H,Mg | + |
| 47 | 4 | KKM-5 | Goalpara | SNB, Dhupdhara | 72-73 | H,Mg | + |
| 48 | 5 | *KKM-6 | Goalpara | SNB, Dhupdhara | 77-78 | H,Gg | - |
| 49 | 6 | KKM-7 | Goalpara | SNB, Dhupdhara | 73-74 | H,Mg | + |
| 50 | 7 | KKM-8 | Goalpara | SNB, Dhupdhara | 72-74 | H,Mg | + |
| 51 | 8 | *KKM-11 | Goalpara | SNB, Dhupdhara | 65-66 | H,Gg | - |
| 52 | 9 | KKM-13 | Goalpara | SNB, Dhupdhara | 67-68 | H,Mg | - |
| 53 | 10 | KKM-14 | Goalpara | SNB, Dhupdhara | 68-69 | H,Mg | + |
| 54 | 11 | KKM-30 | Goalpara | SNB, Dhupdhara | 62-63 | H,Mg | - |
| 55 | 12 | KKM-31 | Goalpara | SNB, Dhupdhara | 61-63 | H,Gg | - |
| 56 | 13 | KKM-32 | Goalpara | SNB, Dhupdhara | 62-63 | H,Mg | + |
| 57 | 14 | KKM-33 | Goalpara | SNB, Dhupdhara | 66-67 | H,Mg | + |
| 58 | 15 | *KKM-34 | Goalpara | SNB, Dhupdhara | 72-73 | H,Gg | - |

**H:** Healthy**, Gg**: Good Growth, **Mg**: Medium growth, **SBN:** Sundarban Nursery, *****disease free sample

**TABLE S4 Primer sequences, expected amplicon size and genetic region amplified in qPCR assays.**

| **Primer name** | **Primer sequence**  **(5’–3’)** | **Amplicon size (bp)** | **Annealing temp. (ᵒC)** | **Genetic region amplified** | **Reference** |
| --- | --- | --- | --- | --- | --- |
| 118F | TGAACGATGTGCGTCAATTG | 190 | 58 | Coat protein gene | Design in this study |
| 118R | TACGTTATGCCCGTAGTGTC |  |  |  |  |
| F BOX F | TCTTGGTTTAGTTTAGTGAAAG | 189 | 58 | Actin | Design in this study |
| F BOX R | ATTCCTTGTCTCAGCTGCTTC |  |  |  |  |

**TABLE S5 Codon Usage Metrics and Genomic Composition Parameters for Citrus Tristeza Virus (CTV) Isolates.**

| **Isolates** | **T3s** | **C3s** | **A3s** | **G3s** | **CAI** | **CBI** | **Fop** | **Nc** | **GC1s** | **GC2s** | **GC12** | **GC3s** | **GC** | **L_sym** | **L_aa** | **Gravy** | **Aromo** |
| --- | --- | --- | --- | --- | --- | --- | --- | --- | --- | --- | --- | --- | --- | --- | --- | --- | --- |
| BHKM-1 | 0.44 | 0.22 | 0.26 | 0.33 | 0.21 | 0.10 | 0.45 | 53.15 | 0.5 | 0.35 | 0.43 | 0.42 | 0.45 | 156 | 160 | -0.3656 | 0.094 |
| BHKM-2 | 0.43 | 0.23 | 0.26 | 0.33 | 0.21 | 0.11 | 0.45 | 53.39 | 0.49 | 0.38 | 0.44 | 0.44 | 0.45 | 156 | 160 | -0.3788 | 0.094 |
| BHKM-3 | 0.44 | 0.22 | 0.26 | 0.33 | 0.21 | 0.12 | 0.46 | 53.92 | 0.49 | 0.38 | 0.44 | 0.43 | 0.45 | 156 | 160 | -0.3788 | 0.094 |
| BHKM-4 | 0.34 | 0.25 | 0.34 | 0.30 | 0.21 | -0.03 | 0.36 | 57.09 | 0.49 | 0.38 | 0.44 | 0.43 | 0.49 | 136 | 140 | -0.3164 | 0.121 |
| BHKM-6 | 0.34 | 0.25 | 0.34 | 0.30 | 0.21 | -0.04 | 0.35 | 57.52 | 0.50 | 0.38 | 0.44 | 0.43 | 0.49 | 136 | 140 | -0.3336 | 0.121 |
| BHKM-7 | 0.34 | 0.25 | 0.34 | 0.30 | 0.22 | -0.03 | 0.36 | 57.09 | 0.49 | 0.38 | 0.44 | 0.43 | 0.49 | 136 | 140 | -0.3164 | 0.121 |
| BHKM-9 | 0.34 | 0.25 | 0.34 | 0.30 | 0.22 | -0.03 | 0.36 | 57.09 | 0.50 | 0.38 | 0.44 | 0.43 | 0.49 | 136 | 140 | -0.3164 | 0.121 |
| DeKM-1 | 0.40 | 0.26 | 0.26 | 0.34 | 0.24 | 0.17 | 0.49 | 56.05 | 0.5 | 0.38 | 0.44 | 0.47 | 0.45 | 217 | 223 | -0.4664 | 0.076 |
| DeKM-2 | 0.44 | 0.24 | 0.26 | 0.35 | 0.24 | 0.15 | 0.48 | 49.29 | 0.49 | 0.38 | 0.43 | 0.44 | 0.44 | 217 | 223 | -0.4574 | 0.076 |
| DeKM-3 | 0.41 | 0.25 | 0.26 | 0.36 | 0.21 | 0.17 | 0.50 | 54.93 | 0.5 | 0.38 | 0.44 | 0.47 | 0.45 | 217 | 223 | -0.4803 | 0.076 |
| DeKM-4 | 0.43 | 0.24 | 0.26 | 0.34 | 0.23 | 0.15 | 0.48 | 49.51 | 0.5 | 0.38 | 0.44 | 0.44 | 0.44 | 217 | 223 | -0.4574 | 0.076 |
| DeKM-5 | 0.43 | 0.24 | 0.26 | 0.35 | 0.24 | 0.14 | 0.48 | 50.13 | 0.5 | 0.38 | 0.43 | 0.45 | 0.45 | 217 | 223 | -0.4574 | 0.076 |
| DeKM-6 | 0.40 | 0.26 | 0.26 | 0.36 | 0.23 | 0.17 | 0.50 | 55.56 | 0.5 | 0.38 | 0.43 | 0.47 | 0.45 | 217 | 223 | -0.4803 | 0.076 |
| DeKM-7 | 0.40 | 0.27 | 0.26 | 0.35 | 0.23 | 0.17 | 0.49 | 56.04 | 0.5 | 0.38 | 0.44 | 0.47 | 0.45 | 217 | 223 | -0.4803 | 0.076 |
| DeKM-8 | 0.44 | 0.25 | 0.27 | 0.34 | 0.24 | 0.14 | 0.48 | 49.3 | 0.5 | 0.38 | 0.44 | 0.45 | 0.45 | 217 | 223 | -0.47 | 0.076 |
| DeKM-9 | 0.42 | 0.25 | 0.25 | 0.35 | 0.22 | 0.15 | 0.48 | 50.16 | 0.5 | 0.38 | 0.44 | 0.46 | 0.45 | 217 | 223 | -0.4534 | 0.076 |
| DeKM-10 | 0.40 | 0.25 | 0.31 | 0.32 | 0.23 | 0.13 | 0.47 | 55.87 | 0.5 | 0.38 | 0.44 | 0.44 | 0.44 | 216 | 223 | -0.4942 | 0.076 |
| GDKM-1 | 0.40 | 0.26 | 0.27 | 0.34 | 0.23 | 0.16 | 0.49 | 56.28 | 0.5 | 0.38 | 0.44 | 0.46 | 0.45 | 217 | 223 | -0.4785 | 0.076 |
| GDKM-2 | 0.40 | 0.27 | 0.28 | 0.34 | 0.23 | 0.13 | 0.48 | 56.83 | 0.5 | 0.38 | 0.44 | 0.46 | 0.44 | 217 | 223 | -0.3220 | 0.085 |
| GDKM-3 | 0.43 | 0.24 | 0.26 | 0.35 | 0.24 | 0.14 | 0.48 | 50.01 | 0.5 | 0.38 | 0.44 | 0.45 | 0.45 | 217 | 223 | -0.4673 | 0.076 |
| GDKM-4 | 0.43 | 0.24 | 0.27 | 0.34 | 0.24 | 0.13 | 0.47 | 50.01 | 0.5 | 0.38 | 0.44 | 0.44 | 0.44 | 217 | 223 | -0.4673 | 0.076 |
| GDKM-5 | 0.40 | 0.26 | 0.27 | 0.34 | 0.24 | 0.15 | 0.48 | 54.64 | 0.5 | 0.38 | 0.44 | 0.46 | 0.45 | 217 | 223 | -0.4888 | 0.076 |
| GDKM- 6 | 0.43 | 0.24 | 0.26 | 0.35 | 0.24 | 0.14 | 0.48 | 50.08 | 0.48 | 0.38 | 0.43 | 0.45 | 0.45 | 217 | 223 | -0.4574 | 0.076 |
| GDKM-8 | 0.42 | 0.25 | 0.25 | 0.36 | 0.23 | 0.15 | 0.48 | 49.5 | 0.5 | 0.38 | 0.44 | 0.46 | 0.45 | 217 | 223 | -0.4673 | 0.076 |
| GDKM-9 | 0.42 | 0.24 | 0.24 | 0.36 | 0.23 | 0.10 | 0.45 | 54.25 | 0.5 | 0.39 | 0.44 | 0.46 | 0.46 | 217 | 223 | -0.3978 | 0.076 |
| GDKM-10 | 0.42 | 0.25 | 0.26 | 0.35 | 0.23 | 0.13 | 0.47 | 50.87 | 0.51 | 0.38 | 0.44 | 0.46 | 0.45 | 217 | 223 | -0.4673 | 0.076 |
| KKM-2 | 0.39 | 0.27 | 0.30 | 0.32 | 0.22 | 0.14 | 0.48 | 56.54 | 0.49 | 0.38 | 0.44 | 0.45 | 0.45 | 216 | 223 | -0.4942 | 0.076 |
| KKM-4 | 0.39 | 0.26 | 0.33 | 0.32 | 0.22 | 0.13 | 0.44 | 56.72 | 0.50 | 0.38 | 0.44 | 0.45 | 0.45 | 216 | 223 | -0.4942 | 0.076 |
| KKM-5 | 0.40 | 0.26 | 0.31 | 0.31 | 0.22 | 0.13 | 0.47 | 57.35 | 0.5 | 0.38 | 0.44 | 0.44 | 0.44 | 216 | 223 | -0.4942 | 0.076 |
| KKM-7 | 0.42 | 0.23 | 0.27 | 0.35 | 0.22 | 0.15 | 0.48 | 52.46 | 0.50 | 0.38 | 0.44 | 0.45 | 0.45 | 217 | 223 | -0.4803 | 0.076 |
| KKM-8 | 0.44 | 0.24 | 0.30 | 0.27 | 0.22 | 0.14 | 0.48 | 58 | 0.49 | 0.39 | 0.44 | 0.39 | 0.43 | 218 | 223 | -0.3399 | 0.099 |
| KKM-14 | 0.43 | 0.24 | 0.26 | 0.34 | 0.22 | 0.15 | 0.48 | 50.5 | 0.5 | 0.38 | 0.44 | 0.45 | 0.45 | 217 | 223 | -0.4704 | 0.076 |
| KKM-32 | 0.40 | 0.26 | 0.26 | 0.36 | 0.22 | 0.18 | 0.50 | 57.03 | 0.50 | 0.38 | 0.44 | 0.48 | 0.45 | 217 | 223 | -0.4848 | 0.076 |
| KKM-33 | 0.40 | 0.26 | 0.26 | 0.35 | 0.22 | 0.17 | 0.49 | 57.01 | 0.49 | 0.38 | 0.43 | 0.47 | 0.45 | 217 | 223 | -0.4834 | 0.076 |
| RTKM-1 | 0.40 | 0.26 | 0.26 | 0.35 | 0.22 | 0.15 | 0.48 | 50.78 | 0.50 | 0.38 | 0.44 | 0.47 | 0.45 | 217 | 223 | -0.4673 | 0.076 |
| RTKM-2 | 0.42 | 0.25 | 0.26 | 0.35 | 0.22 | 0.14 | 0.48 | 51.42 | 0.5 | 0.38 | 0.44 | 0.45 | 0.45 | 217 | 223 | -0.4574 | 0.076 |
| ASKM-1 | 0.43 | 0.24 | 0.26 | 0.35 | 0.24 | 0.14 | 0.48 | 50.13 | 0.5 | 0.38 | 0.44 | 0.44 | 0.44 | 217 | 223 | -0.4574 | 0.076 |
| ASKM-2 | 0.43 | 0.23 | 0.28 | 0.34 | 0.24 | 0.14 | 0.48 | 49.44 | 0.5 | 0.38 | 0.44 | 0.43 | 0.44 | 217 | 223 | -0.4673 | 0.076 |
| MKM-2 | 0.43 | 0.24 | 0.26 | 0.35 | 0.22 | 0.14 | 0.48 | 50.28 | 0.50 | 0.37 | 0.43 | 0.44 | 0.44 | 217 | 223 | -0.4027 | 0.076 |
| KTKM-3 | 0.42 | 0.25 | 0.26 | 0.35 | 0.22 | 0.12 | 0.47 | 54.08 | 0.50 | 0.38 | 0.44 | 0.46 | 0.45 | 217 | 223 | -0.4574 | 0.076 |
| KTKM-4 | 0.42 | 0.25 | 0.27 | 0.34 | 0.22 | 0.11 | 0.46 | 54.31 | 0.51 | 0.38 | 0.45 | 0.45 | 0.45 | 217 | 223 | -0.4552 | 0.072 |
| SRKM-1 | 0.40 | 0.25 | 0.31 | 0.31 | 0.22 | 0.16 | 0.49 | 54.85 | 0.5 | 0.38 | 0.44 | 0.44 | 0.44 | 216 | 223 | -0.4942 | 0.076 |
| SRKM-2 | 0.40 | 0.25 | 0.31 | 0.31 | 0.22 | 0.16 | 0.49 | 54.85 | 0.5 | 0.38 | 0.44 | 0.44 | 0.44 | 216 | 223 | -0.4942 | 0.076 |
| SRKM-4 | 0.41 | 0.25 | 0.26 | 0.36 | 0.22 | 0.14 | 0.48 | 51.43 | 0.49 | 0.38 | 0.44 | 0.46 | 0.45 | 217 | 223 | -0.4475 | 0.076 |
| SRKM-5 | 0.42 | 0.25 | 0.26 | 0.36 | 0.12 | 0.15 | 0.48 | 50.31 | 0.50 | 0.38 | 0.44 | 0.46 | 0.45 | 217 | 223 | -0.4691 | 0.076 |
| SRKM-6 | 0.42 | 0.25 | 0.27 | 0.34 | 0.22 | 0.15 | 0.48 | 49.57 | 0.50 | 0.38 | 0.44 | 0.45 | 0.45 | 217 | 223 | -0.4587 | 0.076 |
| AKM-1 | 0.43 | 0.23 | 0.29 | 0.33 | 0.24 | 0.12 | 0.47 | 50.47 | 0.50 | 0.38 | 0.44 | 0.43 | 0.44 | 217 | 223 | -0.4610 | 0.076 |
| IARI Kpg-3 | 0.40 | 0.25 | 0.31 | 0.31 | 0.23 | 0.15 | 0.48 | 55.09 | 0.5 | 0.38 | 0.44 | 0.43 | 0.44 | 216 | 223 | -0.49 | 0.076 |
| CTV-P2 | 0.41 | 0.25 | 0.32 | 0.29 | 0.26 | 0.08 | 0.47 | 54.120 | 0.5 | 0.38 | 0.44 | 0.42 | 0.44 | 216 | 223 | -0.494 | 0.076 |
| CTV-P3 | 0.43 | 0.24 | 0.26 | 0.34 | 0.23 | 0.02 | 0.43 | 51.630 | 0.5 | 0.38 | 0.44 | 0.44 | 0.45 | 217 | 223 | -0.466 | 0.076 |
| CTV-P4 | 0.43 | 0.24 | 0.26 | 0.34 | 0.23 | 0.03 | 0.44 | 51.680 | 0.49 | 0.38 | 0.43 | 0.44 | 0.44 | 217 | 223 | -0.478 | 0.076 |
| CTV-P7 | 0.41 | 0.25 | 0.32 | 0.29 | 0.26 | 0.08 | 0.47 | 54.120 | 0.5 | 0.38 | 0.44 | 0.42 | 0.44 | 216 | 223 | -0.494 | 0.076 |
| CTV-KAT1 | 0.43 | 0.24 | 0.27 | 0.34 | 0.23 | 0.03 | 0.44 | 52.820 | 0.496 | 0.38 | 0.44 | 0.44 | 0.44 | 217 | 223 | -0.478 | 0.076 |
| CTV-KAT2 | 0.43 | 0.24 | 0.28 | 0.33 | 0.23 | 0.02 | 0.43 | 52.640 | 0.496 | 0.38 | 0.44 | 0.43 | 0.44 | 217 | 223 | -0.478 | 0.076 |
| CTV-KAT3 | 0.43 | 0.24 | 0.28 | 0.33 | 0.23 | 0.02 | 0.43 | 52.510 | 0.491 | 0.38 | 0.44 | 0.43 | 0.44 | 217 | 223 | -0.478 | 0.076 |
| CTV-KAT4 | 0.43 | 0.24 | 0.28 | 0.33 | 0.23 | 0.02 | 0.43 | 52.640 | 0.496 | 0.38 | 0.44 | 0.43 | 0.44 | 217 | 223 | -0.478 | 0.076 |
| CTV-KAT5 | 0.43 | 0.24 | 0.28 | 0.33 | 0.23 | 0.02 | 0.43 | 52.640 | 0.496 | 0.38 | 0.44 | 0.43 | 0.44 | 217 | 223 | -0.478 | 0.076 |
| CTV-KAT6 | 0.43 | 0.23 | 0.27 | 0.34 | 0.23 | 0.03 | 0.44 | 51.710 | 0.496 | 0.38 | 0.44 | 0.44 | 0.44 | 217 | 223 | -0.478 | 0.076 |
| CTV-KAT7 | 0.41 | 0.27 | 0.31 | 0.29 | 0.24 | 0.10 | 0.48 | 57.050 | 0.509 | 0.38 | 0.44 | 0.43 | 0.44 | 217 | 223 | -0.496 | 0.072 |
| CTV-Kpg1 | 0.42 | 0.25 | 0.27 | 0.34 | 0.22 | 0.02 | 0.44 | 54.550 | 0.500 | 0.38 | 0.44 | 0.45 | 0.45 | 216 | 223 | -0.478 | 0.076 |
| CTV-Kpg2 | 0.42 | 0.26 | 0.32 | 0.27 | 0.24 | 0.08 | 0.47 | 53.760 | 0.496 | 0.38 | 0.44 | 0.41 | 0.43 | 217 | 223 | -0.461 | 0.067 |
| CTV-Kpg3 | 0.40 | 0.25 | 0.32 | 0.30 | 0.25 | 0.07 | 0.46 | 55.110 | 0.500 | 0.38 | 0.44 | 0.43 | 0.44 | 216 | 223 | -0.484 | 0.076 |
| CTV-K2 | 0.42 | 0.25 | 0.26 | 0.34 | 0.23 | 0.04 | 0.44 | 53.440 | 0.504 | 0.38 | 0.44 | 0.45 | 0.45 | 216 | 223 | -0.492 | 0.076 |
| CTV-K4 | 0.41 | 0.25 | 0.30 | 0.31 | 0.25 | 0.07 | 0.47 | 58.530 | 0.509 | 0.38 | 0.44 | 0.43 | 0.44 | 217 | 223 | -0.483 | 0.076 |
| CTV-K5 | 0.41 | 0.25 | 0.32 | 0.29 | 0.25 | 0.08 | 0.47 | 57.180 | 0.504 | 0.38 | 0.44 | 0.42 | 0.44 | 217 | 223 | -0.483 | 0.076 |
| CTV-K6 | 0.41 | 0.25 | 0.33 | 0.28 | 0.25 | 0.06 | 0.46 | 54.560 | 0.496 | 0.38 | 0.44 | 0.41 | 0.43 | 216 | 223 | -0.498 | 0.081 |
| CTV-K7 | 0.41 | 0.25 | 0.30 | 0.31 | 0.25 | 0.08 | 0.47 | 57.800 | 0.513 | 0.38 | 0.44 | 0.43 | 0.44 | 217 | 223 | -0.461 | 0.076 |
| CTV-K8 | 0.42 | 0.25 | 0.27 | 0.34 | 0.22 | 0.02 | 0.44 | 54.610 | 0.504 | 0.38 | 0.44 | 0.45 | 0.45 | 216 | 223 | -0.501 | 0.072 |
| CTV-K9 | 0.40 | 0.25 | 0.32 | 0.30 | 0.25 | 0.07 | 0.46 | 55.030 | 0.500 | 0.38 | 0.44 | 0.43 | 0.44 | 216 | 223 | -0.494 | 0.076 |
| CTV-K10 | 0.43 | 0.23 | 0.27 | 0.34 | 0.22 | 0.01 | 0.43 | 52.100 | 0.496 | 0.38 | 0.44 | 0.44 | 0.44 | 217 | 223 | -0.478 | 0.076 |
| CTV-K11 | 0.42 | 0.24 | 0.27 | 0.34 | 0.22 | 0.01 | 0.43 | 54.570 | 0.500 | 0.37 | 0.44 | 0.44 | 0.44 | 216 | 223 | -0.476 | 0.081 |
| CTV-K13 | 0.41 | 0.25 | 0.31 | 0.30 | 0.25 | 0.07 | 0.47 | 57.900 | 0.504 | 0.38 | 0.44 | 0.42 | 0.44 | 217 | 223 | -0.494 | 0.076 |
| CTV-K16 | 0.42 | 0.25 | 0.27 | 0.34 | 0.22 | 0.02 | 0.44 | 54.850 | 0.500 | 0.38 | 0.44 | 0.45 | 0.45 | 216 | 223 | -0.489 | 0.076 |
| CTV-K17 | 0.41 | 0.25 | 0.31 | 0.30 | 0.25 | 0.07 | 0.47 | 57.900 | 0.504 | 0.38 | 0.44 | 0.42 | 0.44 | 217 | 223 | -0.494 | 0.076 |
| CTV-K18 | 0.42 | 0.24 | 0.27 | 0.34 | 0.22 | 0.02 | 0.44 | 54.130 | 0.500 | 0.38 | 0.44 | 0.44 | 0.45 | 216 | 223 | -0.492 | 0.076 |
| CTV-K20 | 0.41 | 0.25 | 0.30 | 0.31 | 0.25 | 0.07 | 0.47 | 57.750 | 0.513 | 0.38 | 0.45 | 0.43 | 0.45 | 217 | 223 | -0.491 | 0.072 |
| CTV-D3 | 0.43 | 0.24 | 0.26 | 0.35 | 0.23 | 0.02 | 0.43 | 51.380 | 0.496 | 0.38 | 0.44 | 0.45 | 0.45 | 217 | 223 | -0.478 | 0.076 |
| CTV-D4 | 0.43 | 0.24 | 0.26 | 0.35 | 0.23 | 0.02 | 0.43 | 51.370 | 0.500 | 0.38 | 0.44 | 0.45 | 0.45 | 217 | 223 | -0.466 | 0.076 |
| CTV-D5 | 0.41 | 0.27 | 0.31 | 0.29 | 0.24 | 0.10 | 0.48 | 57.030 | 0.504 | 0.38 | 0.44 | 0.43 | 0.44 | 217 | 223 | -0.487 | 0.076 |
| CTV-D6 | 0.42 | 0.24 | 0.27 | 0.34 | 0.23 | 0.03 | 0.44 | 51.910 | 0.496 | 0.38 | 0.44 | 0.45 | 0.45 | 217 | 223 | -0.478 | 0.076 |
| CTV-D7 | 0.41 | 0.27 | 0.31 | 0.29 | 0.24 | 0.10 | 0.48 | 57.030 | 0.504 | 0.38 | 0.44 | 0.43 | 0.44 | 217 | 223 | -0.487 | 0.076 |
| CTV-D8 | 0.43 | 0.24 | 0.26 | 0.35 | 0.23 | 0.02 | 0.43 | 51.910 | 0.496 | 0.38 | 0.44 | 0.45 | 0.45 | 217 | 223 | -0.478 | 0.076 |
| CTV-D10 | 0.41 | 0.27 | 0.31 | 0.28 | 0.24 | 0.09 | 0.48 | 56.930 | 0.504 | 0.38 | 0.44 | 0.42 | 0.44 | 217 | 223 | -0.478 | 0.081 |
| CTV-D11 | 0.41 | 0.27 | 0.30 | 0.29 | 0.24 | 0.09 | 0.48 | 57.900 | 0.504 | 0.38 | 0.44 | 0.43 | 0.44 | 217 | 223 | -0.508 | 0.076 |
| CTV-D12 | 0.40 | 0.27 | 0.31 | 0.28 | 0.24 | 0.09 | 0.48 | 57.350 | 0.504 | 0.38 | 0.44 | 0.43 | 0.44 | 217 | 223 | -0.487 | 0.076 |
| CTV-D13 | 0.41 | 0.25 | 0.33 | 0.29 | 0.25 | 0.06 | 0.46 | 54.740 | 0.500 | 0.38 | 0.44 | 0.42 | 0.44 | 216 | 223 | -0.457 | 0.076 |
| CTV-D14 | 0.43 | 0.24 | 0.27 | 0.34 | 0.23 | 0.02 | 0.43 | 52.330 | 0.496 | 0.38 | 0.44 | 0.44 | 0.44 | 217 | 223 | -0.478 | 0.076 |
| CTV-D15 | 0.42 | 0.25 | 0.27 | 0.34 | 0.22 | 0.02 | 0.43 | 54.890 | 0.496 | 0.40 | 0.45 | 0.45 | 0.45 | 216 | 223 | -0.480 | 0.081 |
| CTV-TP1 | 0.41 | 0.26 | 0.31 | 0.29 | 0.23 | 0.07 | 0.46 | 54.080 | 0.496 | 0.38 | 0.44 | 0.43 | 0.44 | 217 | 223 | -0.485 | 0.076 |
| CTV-TP3 | 0.42 | 0.24 | 0.27 | 0.34 | 0.23 | 0.03 | 0.44 | 50.630 | 0.491 | 0.38 | 0.44 | 0.44 | 0.44 | 216 | 223 | -0.500 | 0.081 |
| CTV-TP4 | 0.40 | 0.25 | 0.27 | 0.34 | 0.22 | 0.03 | 0.43 | 57.220 | 0.500 | 0.38 | 0.44 | 0.46 | 0.45 | 217 | 223 | -0.377 | 0.076 |
| CTV-TP5 | 0.42 | 0.24 | 0.28 | 0.34 | 0.22 | 0.02 | 0.43 | 53.110 | 0.496 | 0.37 | 0.43 | 0.44 | 0.44 | 216 | 223 | -0.471 | 0.076 |
| CTV-TP6 | 0.38 | 0.27 | 0.28 | 0.34 | 0.23 | 0.03 | 0.44 | 55.600 | 0.496 | 0.38 | 0.44 | 0.47 | 0.45 | 217 | 223 | -0.473 | 0.072 |
| CTV-TP7 | 0.41 | 0.25 | 0.27 | 0.34 | 0.22 | 0.02 | 0.43 | 55.850 | 0.509 | 0.38 | 0.44 | 0.45 | 0.45 | 216 | 223 | -0.482 | 0.072 |
| CTV-TP9 | 0.41 | 0.25 | 0.27 | 0.34 | 0.22 | 0.00 | 0.42 | 59.060 | 0.500 | 0.38 | 0.44 | 0.46 | 0.45 | 217 | 223 | -0.481 | 0.076 |
| CTV-TP11 | 0.41 | 0.26 | 0.30 | 0.30 | 0.24 | 0.08 | 0.47 | 53.650 | 0.500 | 0.38 | 0.44 | 0.43 | 0.44 | 217 | 223 | -0.511 | 0.072 |
| CTV-TP12 | 0.42 | 0.24 | 0.27 | 0.34 | 0.23 | 0.02 | 0.43 | 56.580 | 0.496 | 0.38 | 0.44 | 0.44 | 0.45 | 217 | 223 | -0.478 | 0.076 |
| AR2 | 0.41 | 0.25 | 0.31 | 0.30 | 0.25 | 0.07 | 0.47 | 58.610 | 0.509 | 0.38 | 0.44 | 0.42 | 0.44 | 217 | 223 | -0.483 | 0.076 |
| AR13 | 0.41 | 0.25 | 0.32 | 0.29 | 0.24 | 0.04 | 0.44 | 57.480 | 0.500 | 0.38 | 0.44 | 0.42 | 0.44 | 217 | 223 | -0.492 | 0.076 |
| AR16 | 0.41 | 0.25 | 0.31 | 0.30 | 0.25 | 0.07 | 0.47 | 58.140 | 0.509 | 0.38 | 0.44 | 0.42 | 0.44 | 217 | 223 | -0.497 | 0.076 |
| MB3 | 0.42 | 0.24 | 0.28 | 0.33 | 0.23 | 0.04 | 0.44 | 50.250 | 0.491 | 0.38 | 0.44 | 0.44 | 0.44 | 216 | 223 | -0.500 | 0.081 |
| MB7 | 0.42 | 0.25 | 0.27 | 0.34 | 0.23 | 0.03 | 0.44 | 50.300 | 0.500 | 0.38 | 0.44 | 0.45 | 0.45 | 217 | 223 | -0.457 | 0.076 |
| AG26 | 0.42 | 0.24 | 0.34 | 0.27 | 0.27 | 0.09 | 0.47 | 56.430 | 0.509 | 0.38 | 0.44 | 0.40 | 0.43 | 217 | 223 | -0.475 | 0.081 |
| AG28 | 0.43 | 0.23 | 0.29 | 0.34 | 0.21 | -0.02 | 0.41 | 52.890 | 0.496 | 0.38 | 0.44 | 0.43 | 0.44 | 216 | 223 | -0.485 | 0.076 |
| N1 | 0.41 | 0.26 | 0.30 | 0.31 | 0.25 | 0.07 | 0.47 | 59.200 | 0.504 | 0.38 | 0.44 | 0.43 | 0.44 | 217 | 223 | -0.452 | 0.076 |
| Mnp1 | 0.43 | 0.24 | 0.26 | 0.36 | 0.23 | 0.04 | 0.45 | 50.240 | 0.491 | 0.38 | 0.43 | 0.45 | 0.44 | 217 | 223 | -0.472 | 0.081 |
| Mnp2 | 0.42 | 0.25 | 0.28 | 0.34 | 0.23 | 0.02 | 0.43 | 54.640 | 0.504 | 0.38 | 0.44 | 0.45 | 0.45 | 217 | 223 | -0.482 | 0.076 |
| Mnp3 | 0.42 | 0.24 | 0.27 | 0.34 | 0.22 | 0.01 | 0.43 | 54.060 | 0.504 | 0.38 | 0.44 | 0.45 | 0.45 | 217 | 223 | -0.464 | 0.076 |
| VT | 0.42 | 0.25 | 0.25 | 0.36 | 0.22 | 0.02 | 0.43 | 55.530 | 0.491 | 0.38 | 0.43 | 0.46 | 0.45 | 217 | 223 | -0.500 | 0.076 |
| T30 | 0.45 | 0.22 | 0.30 | 0.31 | 0.23 | 0.08 | 0.47 | 51.290 | 0.491 | 0.38 | 0.43 | 0.41 | 0.43 | 217 | 223 | -0.501 | 0.076 |
| T36 | 0.42 | 0.26 | 0.30 | 0.30 | 0.24 | 0.08 | 0.47 | 54.550 | 0.487 | 0.38 | 0.43 | 0.43 | 0.43 | 217 | 223 | -0.493 | 0.072 |
| T3 | 0.42 | 0.24 | 0.27 | 0.34 | 0.23 | 0.03 | 0.44 | 51.630 | 0.500 | 0.38 | 0.44 | 0.44 | 0.44 | 217 | 223 | -0.478 | 0.076 |
| B165 | 0.40 | 0.26 | 0.26 | 0.35 | 0.23 | 0.06 | 0.46 | 50.840 | 0.491 | 0.38 | 0.43 | 0.47 | 0.45 | 217 | 223 | -0.481 | 0.076 |
| **NZRB-G90** | 0.41 | 0.27 | 0.31 | 0.28 | 0.24 | 0.09 | 0.48 | 55.630 | 0.491 | 0.38 | 0.43 | 0.42 | 0.44 | 217 | 223 | -0.473 | 0.072 |
| **Average** | **0.414** | **0.247** | **0.283** | **0.327** | **0.229** | **0.080** | **0.456** | **54.042** | **0.499** | **0.377** | **0.438** | **0.441** | **0.446** | **212.342** | **218.430** | **-0.466** | **0.078** |
| **Standard Deviation** | **0.018** | **0.012** | **0.025** | **0.024** | **0.016** | **0.057** | **0.028** | **2.757** | **0.006** | **0.005** | **0.005** | **0.016** | **0.010** | **17.512** | **18.036** | **0.042** | **0.009** |
| **Standard Error** | **0.002** | **0.002** | **0.003** | **0.003** | **0.002** | **0.008** | **0.004** | **0.372** | **0.001** | **0.001** | **0.001** | **0.002** | **0.001** | **2.361** | **2.432** | **0.006** | **0.001** |

**TABLE S6 Relative codon usage bias (RCUB) of mild and severe Strains of CTV concerning the Host.**

CODONS UUU UUC UUA UUG CUU CUC CUA CUG AUU AUC AUA GUU GUC GUA GUG UCU UCC UCA UCG AGU AGC CCU CCC CCA CCG ACU ACC ACA ACG GCU GCC GCA GCG UAU UAC CAU CAC CAA CAG AAU AAC AAA AAG GAU GAC GAA GAG UGU UGC CGU CGC CGA CGG AGA AGG GGU GGC GGA GGG

Cr(Host) 1.08 0.92 0.88 1.28 0.91 0.43 0.98 1.48 0.9 0.63 1.34 0.48 0.54 1.29 1.41 0.84 1.33 0.69 0.87 0.85 1.54 0.69 1.29 0.49 1.49 0.84 1.21 0.45 1.41 1.01 1.13 0.46 1.03 0.97 1.05 0.95 1 1 1.11 0.89 0.93 1.07 1.33 0.67 0.99 1.01 0.76 1.24 0.91 0.7 0.69 0.56 1.84 1.29 1.09 0.98 1.21 0.71 1

DeKM-5 1.129 0.571 0.923 2.231 1.154 0 0.231 0.462 0.429 0.857 1.714 1.067 1.333 0.533 1.067 1.273 0 1.091 0 1.636 0 1 0 0 1.23 1.867 0.8 0.533 0.8 1.474 1.053 0.632 0.842 1.25 0.75 0.667 1.333 1.25 0.75 0.8 1.2 0.941 1.059 1.238 0.762 1.111 0.889 2 0 1.308 0.462 0.923 0.462 0.923 0.923 1.5 1 0.5 1

IARI_Kpg-3 1.429 0.571 1.846 2.308 1.154 0 0 0.692 1 0.5 1.5 1.333 1.067 0.533 1.067 2.727 0.545 0 1.091 1.091 0.545 1.6 0 0.8 1.6 1.333 1.067 0.8 0.8 1.647 0.706 1.412 0.235 1 1 0 2 1 1 0.4 1.6 1.176 0.824 1.2 0.8 1 1 2 0 2.308 0.462 0.923 0.462 1.385 0.462 1.882 0.471 0.706 0.941
